# Screen Time Is Associated With Cardiometabolic and Cardiovascular Disease Risk in Childhood and Adolescence

**DOI:** 10.1161/JAHA.125.041486

**Published:** 2025-08-06

**Authors:** David Horner, Marie Jahn, Klaus Bønnelykke, Bo Chawes, Trine Flensborg‐Madsen, Ann‐Marie Malby Schoos, Jakob Stokholm, Morten Arendt Rasmussen

**Affiliations:** ^1^ COPSAC, Copenhagen Prospective Studies on Asthma in Childhood Herlev and Gentofte Hospital, University of Copenhagen Denmark; ^2^ Unit of Medical Psychology, Department of Public Health University of Copenhagen Denmark; ^3^ Department of Pediatrics Slagelse Hospital Slagelse Denmark; ^4^ Department of Food Science, Section of Food Microbiology and Fermentation University of Copenhagen Denmark; ^5^ National Institute of Public Health University of Southern Denmark Copenhagen Denmark

**Keywords:** adolescence, cardiometabolic risk, metabolomics, screen time, sleep, Risk Factors, Pediatrics, Obesity, Cardiovascular Disease

## Abstract

**Background:**

Screen time in children and adolescents may be linked to cardiometabolic and cardiovascular risk.

**Methods:**

We analyzed data from >1000 participants in the COPSAC2010 and COPSAC2000 (Copenhagen Prospective Studies on Asthma in Childhood) mother–child 2000 and 2010 cohorts. Discretionary screen time, reported by parents or self, was assessed in relation to a composite cardiometabolic risk score based on *Z* scores of waist circumference, systolic blood pressure, high‐density lipoprotein cholesterol, triglycerides, and glucose. Secondary outcomes included insulin resistance, inflammation, lipoproteins, and anthropometry. A predicted cardiovascular risk score, derived from Cox models trained on UK Biobank data, was also assessed as an outcome. We evaluated whether lifestyle factors (sleep, physical activity, diet, puberty) moderated these associations. Blood nuclear magnetic resonance metabolomics were modeled using supervised machine learning to identify a metabolic screen‐time signature.

**Results:**

Each additional hour of screen time was associated with higher cardiometabolic risk in both children (β=0.08 [0.01–0.14], *P*=0.021) and adolescents (β=0.13 [0.07–0.20], *P*=0.001). Sleep duration significantly moderated this association in both cohorts (childhood: *P*=0.029; adolescence: *P*=0.012), with higher risk among those with shorter sleep. Screen time was also associated with higher predicted cardiovascular risk in adolescence (β=0.07 [0.01–0.13], *P*=0.017). A screen time‐associated metabolomic signature identified in childhood was validated in adolescence (β=0.14 [0.03–0.26], *P*=0.014).

**Conclusions:**

Screen time was positively associated with cardiometabolic and cardiovascular risk, and these associations were stronger among children and adolescents with shorter sleep duration. These findings highlight the importance of jointly considering screen time and sleep patterns in the assessment of early‐life risk factors for cardiometabolic and cardiovascular health.

**Nonstandard Abbreviations and Acronyms**
CMR: cardiometabolic risk
COPSAC2000: Copenhagen Prospective Studies on Asthma in Childhood mother–child cohort 2000
COPSAC2010: Copenhagen Prospective Studies on Asthma in Childhood mother–child cohort 2010
SBP: systolic blood pressure

**Research Perspective**

**What Is New?**

- Screen time in childhood and adolescence is positively associated with higher cardiometabolic risk, supported by consistent associations with metabolic syndrome components, insulin resistance, inflammation, and adverse lipid profiles.
- Sleep duration moderates these associations; shorter sleep amplifies the relationship between screen time and cardiometabolic risk in both children and adolescents.
- A supervised machine‐learning model identified a robust blood‐based metabolic signature of screen time, and screen time was also associated with a higher predicted cardiovascular risk score in adolescence using blood metabolomics.

**What Question Should Be Addressed Next?**

- Can targeted behavioral interventions aimed at reducing screen time and improving sleep duration mitigate cardiometabolic and cardiovascular risk profiles during critical developmental windows?

Cardiovascular disease (CVD) is a leading cause of morbidity and mortality worldwide and has its roots in childhood.^1^ Key predictors of early onset of CVD are the presence of cardiometabolic risk (CMR) factors,^2^ such as components of the metabolic syndrome, insulin resistance, inflammation, apolipoprotein B‐containing lipoproteins, and obesity.^3^, ^4^

As we transition into the digital age, these risks may be exacerbated, because children and adolescents are spending an increasing amount of time engaged with screens and digital content, be it for educational, recreational, or social purposes. Emerging research has begun to shed light on the potential health implications of this trend, with several studies noting associations between screen time and CMR factors in childhood and adolescence.^5^, ^6^, ^7^ However, the relationship between screen time and CMR is complex and likely multifactorial. Reductions in sleep duration,^8^ increased sedentary time,^9^ reduced physical activity,^10^ and unhealthy dietary patterns^11^ have all been associated with increased screen time. It is plausible that they may serve as moderators, or mediators, in the relationship between screen time and CMR.^12^ This interplay is important to investigate, because prospective studies show that increased screen time in adolescence is associated with a higher risk of obesity, elevated waist circumference, and diabetes in adulthood.^6^ Previous studies investigating this relationship have lacked objective measures for contextual lifestyle factors, relying on subjective self‐reported measures of diet, sleep, and physical activity, and thus may be subject to bias and inaccuracies.^13^

In this study, we aimed to address these limitations by assessing longitudinal associations between screen time and CMR factors in 2 mother–child cohorts. By juxtaposing the 2 cohorts from the COPSAC (Copenhagen Prospective Studies on Asthma in Childhood), we gain valuable insights into how screen time patterns and their potential impact on CMR evolve from childhood through adolescence. We hypothesize that higher screen time during childhood and adolescence is associated with adverse CMR, as defined by the components of the metabolic syndrome. By using targeted blood metabolomics, we identify a metabolic signature of screen time, showing that screen time‐associated metabolic disturbances are robust predictors across cohorts. Finally, we link screen time to CVD outcomes using a nuclear magnetic resonance (NMR) CVD score trained in a large prospectively followed adult cohort.

## METHODS

### Data Availability Statement and Ethics Statement

The data that support the findings of this study are available from the corresponding author upon reasonable request. The study was conducted in accordance with the Declaration of Helsinki and was approved by the Danish Ethics Committee (H‐B‐2008‐093) and the Danish Data Protection Agency (2015‐41‐3696). The study was conducted and monitored in accordance with the requirements of Good Clinical Practice as defined in guidelines, EU Clinical Trials Directive (2001/20/EC), and EU GCP Directive (2005/28/EC). All study participants signed approved informed consent forms before any study‐related procedures. The confidentiality of all study participants is protected in accordance with Good Clinical Practice guidelines.

### Study Design

COPSAC2010 (Copenhagen Prospective Studies on Asthma in Childhood mother–child cohort 2010) is a prospective general population study that consists of 700 mother–child pairs with extensive phenotyping from 14 clinical visits and exposure assessments since birth, up to the age of 10 years.^14^ COPSAC2000 (Copenhagen Prospective Studies on Asthma in Childhood mother–child cohort 2010) is likewise a prospective mother–child cohort consisting of 411 children born of asthmatic mothers with extensive phenotyping from 19 clinical visits and exposure assessments since birth, up to the age of 18 years.^15^ Participants in COPSAC2010 (n=2) and COPSAC2000 (n=1) with type 1 diabetes were excluded from the analysis due to potential confounding.^16^

### Screen Time Measurement

Screen time, the primary exposure, was measured using questionnaire responses from the COPSAC2010 and COPSAC2000 cohorts, focusing on discretionary screen time. In COPSAC2010, parents reported children's average screen time at age 6 and 10 years on weekdays and weekends. In COPSAC2000, 18‐year‐old participants detailed screen time from Monday to Thursday and Friday to Sunday, differentiated by type. Total screen time was calculated, and weighted averages were derived. Further details are in Data S1.

### Cardiometabolic Outcomes

Our primary outcome was a CMR score derived from the components of the metabolic syndrome.^17^, ^18^ Before calculating CMR scores at each clinical visit, the 5 measures (waist circumference, systolic blood pressure [SBP], high‐density lipoprotein [HDL] cholesterol, triglycerides, and glucose) were adjusted for sex and age; SBP was further adjusted for height.^19^ The total CMR score was then calculated by adding internally (within each cohort) standardized *Z* scores of waist circumference, SBP, negative HDL cholesterol, logged triglycerides, and glucose, and then dividing the sum by the square root of 5.^17^, ^18^

Secondary outcomes included other established CMR risk factors including hemoglobin A1c, Homeostatic Model Assessment for Insulin Resistance, high‐sensitivity C‐reactive protein, glycoprotein acetyls, apolipoprotein B, and numerous measurements of body anthropometrics. Furthermore, we included an NMR cardiovascular risk score based on sex‐stratified Cox proportional hazards models for 10‐year CVD risk trained in the UK Biobank.^20^ Our analysis used the sex‐stratified model coefficients from this work by internally *Z*‐scoring the individual model components in the COPSAC cohorts, multiplying them by the model coefficients, and summing these components into a total CVD risk score. We subsequently *Z*‐scored for each of the respective COPSAC cohorts to facilitate interpretation of estimates. Nightingale targeted blood NMR data were available at age 10 years for COPSAC2010 and age 18 years for COPSAC2000. Further details are in Data S1.

### Body Anthropometrics

Anthropometrics were assessed at each clinical visit. Body composition analysis at age 10 years in COPSAC2010 and age 18 years in COPSAC2000 was measured by bioelectrical impedance analysis using a Tanita scale (Health Monitor, version 3.2.7) to derive muscle mass (kilograms), fat mass (kilograms), and bone mass (kilograms). Fat‐free mass (body weight−fat mass), fat mass index (fat mass/[height^2^]), and fat‐free mass index (fat free mass/[height^2^ {meters}]) were derived from these measurements. Further details are in Data S1.

### Accelerometer‐Derived Sleep and Activity Data

Activity and sleep information were derived from accelerometry data (Actigraph GT3X+, 30 Hz) over 14 days using the GGIR package in R (version 2.9.0).^21^, ^22^, ^23^, ^24^

To account for individual variations in calibration between accelerometers, we used the autocalibration feature of the GGIR package to calibrate measurements from individual accelerometers. As a quality control measure, we removed measurements for 15 individuals in COPSAC2010, and 7 in COPSAC2000 whose data were unable to autocalibrate. Participant data were excluded from analysis if they had <4 days of data, and within individuals, days of measurement were removed if they had <16 hours of wear time.

Mean daily vector magnitude (milli‐G) over 5‐second epochs were used to calculate sedentary time (time spent at <30 milli‐G), light activity (time spent between 30 and 100 milli‐G), moderate activity (time spent between 100 and 400 milli‐G), and vigorous activity times (time spent >400 milli‐G).^23^ Sleep onset was defined as the average detected onset of sleep, and sleep duration was the average length of sleep (hours).^22^

### Covariates

The analysis was conducted unadjusted and fully adjusted. Unadjusted analysis covariates included only sex and age, because there are well‐documented sex and age‐dependent effects on our outcome measures.^25^, ^26^ Fully adjusted multivariable analysis in both cohorts were further adjusted for social circumstances (principal component 1 of a principal component analysis of household income, maternal education level, and maternal age), maternal smoking during pregnancy, number of siblings, and accelerometer‐derived sedentary time, symptoms of attention‐deficit/ hyperactivity disorder, light activity time, moderate to vigorous activity time (a sum of moderate and vigorous activity time), sleep duration, and time of sleep onset. In COPSAC2010, we included further adjustment for objective markers of pubertal progression using the gonadotropic hormones, luteinizing hormone, and follicle‐stimulating hormone, which increase at puberty onset.^27^ Luteinizing hormone and follicle‐stimulating hormone were included in models as an interaction effect with child sex to account for potential differences between sexes. Additionally, in COPSAC2010, adjustments were made for dietary patterns assessed at the age of 10 years. Further details are in Data S1.

### Statistical Analysis

Linear regression models were used to assess the associations of screen time on CMR factors and anthropometric outcomes. Because we had repeated measurements of outcome data at age 6 and 10 years in COPSAC2010, we used linear mixed models from the lme4 R package (version 1.1.28) with a fixed slope and variable intercept for our primary outcome analyses.^28^ We tested interaction terms in fully adjusted models with sex, based on known biological and developmental variation in cardiometabolic risk. We explored the potential for modifiable lifestyle factors such as sleep, physical activity measures, and dietary patterns to moderate the association of screen time on CMR. Mediation analyses were performed only in instances where Gaussian graphical models identified a plausible indirect pathway between screen time and CMR. All interaction models and mediation models were multivariable. The mediation package in R was used for mediation analysis (version 4.5.0).^29^ In our models for CMR outcomes, we a priori decided not to include a child's anthropometrics as covariates. This was based on the potential for anthropometrics to act as a causal intermediary between screen time and CMR outcomes.

Gaussian graphical models were used to illustrate the non‐0 relationships (95% Cl) between screen time, covariates, and total CMR, controlling for the linear effects of all covariates expressed as partial correlations.

To further explore the relationship between screen time and its metabolic associations between cohorts, we used supervised machine learning modeling, sparse partial least squares. The sparse partial least squares model was trained in COPSAC2010 using the Nightingale Health high‐throughput targeted NMR‐metabolomics platform as the predictor and screen time as the outcome variable.^30^ Screen time was *Z*‐scored relative to each cohort for this analysis, allowing comparison of estimates.

A 2‐sided *P*<0.05 was considered statistically significant. All statistical analyses were performed using R (version 4.1.1). Further details are in Data S1.

## RESULTS

### Baseline Characteristics

For the COPSAC2010 cohort, screen time was available for 657 children (94.1%) at age 6 years and for 630 children (90.3%) at age 10 years. For COPSAC2000, screen time was available for 364 adolescents (88.6%). Marked differences in screen time were observed between the cohorts. Average screen time in COPSAC2010 was 2.0±0.9 hours at age 6 years and 3.2±1.2 hours at age 10 years, representing a significant increase over time (*P*<0.001). COPSAC2000 at age 18 years had a significantly higher average screen time of 6.1±2.1 hours (*P*<0.001) (Figure S1). Baseline characteristic differences between the cohorts included parental income, maternal education, maternal age at birth, gestational age, maternal smoking in pregnancy, and number of siblings (*P*<0.001) (Table S1).

In COPSAC2010, screen time was positively associated with age, male sex, sedentary time, sleep onset time, attention‐deficit/hyperactivity disorder symptoms, and a Western dietary pattern, and negatively associated with light and moderate activity time and sleep duration (Table S2, dietary patterns illustrated in Figure S2). Similarly, in COPSAC2000, screen time had significant positive associations with male sex and maternal smoking during pregnancy and was negatively associated with social circumstances and sleep duration (Table S2). Moreover, with respect to sex differences, boys in COPSAC2010 used more screen time at age 10 years compared with girls (3.4 versus 3.0, *P*<0.001), whereas no significant difference was noted at age 6 years (*P*=0.196). In COPSAC2000, a similar sex difference in screen time was noted (6.6 versus 5.7, *P*<0.001). Both cohorts demonstrated numerous significant sex‐related differences across CMR profiles, anthropometrics, and covariates (Table S3).

Between cohorts, differences in covariates such as age, maternal smoking, sedentary time, physical activity, and sleep patterns were identified (*P*<0.001). All CMR factors were significantly different between cohorts (*P*<0.001), except for apolipoprotein B. We computed a total CMR score by summing *Z* scores of waist circumference, SBP, HDL cholesterol, triglycerides, and glucose levels^18^ in both cohorts. Cohort differences were noted for all measures of body anthropometrics (*P*<0.001) (Table 1). Within cohorts, correlations between screen time and model covariates (Figure S3), and correlations between CMR factors (Figure S4) are further visualized in comprehensive heatmaps.

**Table 1:** Exposure, Outcome, and Covariate Data for COPSAC2010 and COPSAC2000

COPSAC2010 and COPSAC2000 comparison	COPSAC2010	COPSAC2000	C2010/C2000 % missing
No.	630	364	…
Screen time and cardiometabolic outcomes			
Average screen time, h, mean±SD	3.20±1.21	6.11±2.11	0/0
Waist size, cm, mean±SD	64.75±7.42	80.50±11.34	4.8/1.1
Systolic blood pressure, mean±SD	103.94±6.93	115.78±9.99	5.6/1.4
HDL mmol/L, mean±SD	1.56±0.32	1.22±0.26	20.0/8.0
Triglyceride, mmol/L, mean±SD	0.74±0.32	0.97±0.44	19.8/8.0
Glucose, mmol/L, mean±SD	4.18±0.36	5.10±0.40	16.8/8.0
HbA1c, mmol/mol, mean±SD	32.10±2.68	31.36±2.80	19.2/8.8
HOMA‐IR, mean±SD	1.96±0.25	2.78±1.92	20.8/9.3
High sensitivity CRP, mg/L, mean±SD	0.57±1.54	1.71±2.55	46.3/10.2
GlycA, mmol/L, mean±SD	0.68±0.07	0.76±0.11	16.8/17.9
ApoB, g/L, mean±SD	0.69±0.12	0.68±0.15	16.8/17.9
Cohort characteristics and covariates			
Male sex, n (%)	324 (51.4)	179 (49.2)	0
Age, y, mean±SD	10.30±0.39	17.73±0.57	0
Siblings, mean±SD	1.47±0.94	1.24±0.87	0
Maternal pregnancy smoking, n (%)	45 (7.1)	83 (22.8)	0
Sedentary time, h, mean±SD	8.47±1.20	10.91±1.31	23.8/22.8
Light activity time, h, mean±SD	3.84±0.66	3.52±0.75	23.8/22.8
Moderate activity time, h, mean±SD	2.15±0.53	1.59±0.55	23.8/22.8
Vigorous activity time, h, mean±SD	0.42±0.21	0.09±0.10	23.8/22.8
Sleep, h, mean±SD	9.04±0.76	7.73±0.86	23.8/22.8
Sleep onset time, h, mean±SD	21.84±0.82	24.46±1.19	23.8/22.8
No. of valid days (accelerometer), mean±SD	12.32±1.66	11.70±2.13	23.8/22.8
Body anthropometrics			
BMI, mean±SD	17.08±2.38	22.91±4.10	11.1/2.7
Body weight, kg, mean±SD	35.77±6.98	70.14±14.06	10.8/2.2
Fat mass, kg, mean±SD	7.99±3.24	16.97±8.28	11.1/2.7
Fat percent, mean±SD	21.71±4.78	23.70±8.22	11.1/2.7
Muscle mass, kg, mean±SD	26.31±4.10	50.46±9.71	11.1/2.7
Skeletal mass, kg, mean±SD	15.73±2.44	30.26±5.93	11.1/2.7
Bone mass, kg, mean±SD	1.48±0.22	2.68±0.48	11.1/2.7
Fat‐free mass, kg, mean±SD	27.79±4.31	53.14±10.19	11.1/2.7
Fat mass index, mean±SD	3.80±1.38	5.66±2.92	11.3/2.7
Fat‐free mass index, mean±SD	13.28±1.22	17.28±2.14	11.3/2.7

This table presents the baseline characteristics, outcomes, and covariates for the COPSAC2010 (n=630) and COPSAC2000 (n=364) cohorts. It includes data on screen time, cardiometabolic risk factors, anthropometrics, and lifestyle behaviors such as physical activity and sleep patterns. Significant differences between the cohorts are indicated, including differences in screen time, cardiometabolic risk factors, and anthropometrics. The data presented in this table provide a comprehensive overview of the cohorts' characteristics. All between‐cohort comparisons were statistically significant (*P*<0.001), except for apoB (*P*=0.207) and sex distribution (*P*=0.536). ApoB indicates apolipoprotein B; BMI, body mass index; COPSAC2000, Copenhagen Prospective Studies on Asthma in Childhood mother–child cohort 2000; COPSAC2010, Copenhagen Prospective Studies on Asthma in Childhood mother–child cohort 2010; CRP, C‐reactive protein; GlycA, glycoprotein acetyls; HbA1c, hemoglobin A1c; HDL, high‐density lipoprotein; and HOMA‐IR, Homeostasis Model Assessment of Insulin Resistance.

### Screen Time Is Associated With Cardiometabolic Risk in COPSAC2010

In COPSAC2010, we used mixed models to assess the association between screen time and total CMR and its constituent components at age 6 and 10 years. After adjusting for relevant confounders, there was a significant positive association between screen time (per hour increase) and CMR (0.08 [0.01–0.14], *P*=0.021) (Table 2). In boys, the association for CMR (0.10 [0.02–0.19], *P*=0.013) was directionally stronger than in girls (0.02 [−0.08 to 0.12], *P*=0.694), but there was no significant interaction between sexes (Table S4). Cross‐sectionally at age 10 years, screen time associations with CMR were stronger than at age 6 years (0.16 [0.05–0.27], *P*=0.007 versus 0.06 [−0.06 to 0.17], *P*=0.321) (Table 2, Table 3). Further adjustment for the COPSAC2010 prenatal interventions with ω‐3 long‐chain polyunsaturated fatty acids and high‐dose vitamin D did not alter these associations (Table S5).

**Table 2:** Associations Between Screen Time and Cardiometabolic Risk Factors in COPSAC2010 and COPSAC2000

COPSAC2010 age 6 and 10 y mixed model	No.	Unadjusted [95% Cl] (*P* value)	Adjusted [95% Cl] (*P* value)
Cardiometabolic risk (*Z* score)	513/525	0.08 [0.02 to 0.15] (*P*=0.009)	0.08 [0.01 to 0.14] (*P*=0.021)
Waist size, cm	621/600	0.22 [−0.08 to 0.52] (*P*=0.157)	0.2 [−0.11 to 0.5] (*P*=0.21)
Systolic blood pressure, mm Hg	605/595	0.09 [−0.27 to 0.45] (*P*=0.621)	0.06 [−0.31 to 0.42] (*P*=0.758)
HDL cholesterol, mmol/L	491/504	−0.02 [−0.04 to 0] (*P*=0.027)	−0.02 [−0.03 to 0] (*P*=0.052)
Triglycerides, mmol/L* (logged)	465/505	0.03 [0.01 to 0.06] (*P*=0.006)	0.03 [0.01 to 0.06] (*P*=0.013)
Glucose, mmol/L†	507/509	0 [−0.03 to 0.03] (*P*=0.998)	0 [−0.03 to 0.03] (*P*=0.908)

COPSAC2010 age 10 y linear model	No.	Unadjusted [95% Cl] (*P* value)	Adjusted [95% Cl] (*P* value)
Cardiometabolic risk (*Z* score)	525	0.14 [0.06 to 0.22] (*P*=0.001)	0.12 [0.03 to 0.2] (*P*=0.011)
Waist size, cm	600	0.47 [−0.03 to 0.98] (*P*=0.068)	0.34 [−0.19 to 0.88] (*P*=0.211)
Systolic blood pressure, mm Hg	595	0.19 [−0.29 to 0.66] (*P*=0.446)	0.19 [−0.32 to 0.7] (*P*=0.458)
HDL cholesterol, mmol/L	504	−0.03 [−0.05 to 0] (*P*=0.037)	−0.02 [−0.05 to 0] (*P*=0.091)
Triglycerides, mmol/L* logged	505	0.06 [0.03 to 0.08] (*P*<0.001)	0.05 [0.02 to 0.07] (*P*=0.003)
Glucose, mmol/L	524	0.01 [−0.02 to 0.04] (*P*=0.494)	0.01 [−0.02 to 0.04] (*P*=0.467)
HbA1c, mmol/mol	509	0.01 [−0.19 to 0.21] (*P*=0.928)	0 [−0.21 to 0.21] (*P*=0.987)
HOMA‐IR	499	0.02 [0 to 0.04] (*P*=0.013)	0.02 [0 to 0.04] (*P*=0.024)
High‐sensitivity CRP, mg/L	338	0.03 [−0.12 to 0.17] (*P*=0.724)	−0.02 [−0.17 to 0.13] (*P*=0.827)
GlycA, mmol/L	524	0.01 [0 to 0.01] (*P*=0.01)	0.01 [0 to 0.01] (*P*=0.053)
ApoB, g/L	524	0 [−0.01 to 0.01] (*P*=0.927)	0 [−0.01 to 0.01] (*P*=0.961)
NMR cardiovascular risk score (*Z* score)	520	0.07 [0 to 0.14] (*P*=0.054)	0.06 [−0.02 to 0.13] (*P*=0.15)

COPSAC2000 age 18 y linear model	No.	Unadjusted [95% Cl] (*P* value)	Adjusted [95% Cl] (*P* value)
Cardiometabolic risk (*Z* score)	335	0.14 [0.08 to 0.2] (*P*<0.001)	0.13 [0.07 to 0.2] (*P*<0.001)
Waist size, cm	360	1.47 [0.93 to 2.01] (*P*<0.001)	1.3 [0.76 to 1.84] (*P*<0.001)
Systolic blood pressure, mm Hg	359	0.7 [0.23 to 1.16] (*P*=0.004)	0.63 [0.15 to 1.11] (*P*=0.011)
HDL cholesterol, mmol/L	335	−0.01 [−0.03 to 0] (*P*=0.06)	−0.01 [−0.03 to 0] (*P*=0.032)
Triglycerides, mmol/L* logged	335	0.02 [0 to 0.04] (*P*=0.042)	0.02 [0 to 0.04] (*P*=0.103)
Glucose, mmol/L	335	0 [−0.02 to 0.02] (*P*=0.675)	0.01 [−0.02 to 0.03] (*P*=0.611)
HbA1c, mmol/mol	333	−0.08 [−0.23 to 0.07] (*P*=0.282)	−0.09 [−0.24 to 0.06] (*P*=0.24)
HOMA‐IR	330	0.09 [−0.01 to 0.19] (*P*=0.064)	0.08 [−0.02 to 0.18] (*P*=0.13)
High‐sensitivity CRP, mg/L	327	0.04 [−0.09 to 0.17] (*P*=0.541)	0.01 [−0.13 to 0.15] (*P*=0.863)
GlycA, mmol/L	299	0.01 [0.01 to 0.02] (*P*<0.001)	0.01 [0.01 to 0.02] (*P*<0.001)
ApoB, g/L	299	0.01 [0.01 to 0.02] (*P*=0.001)	0.01 [0.01 to 0.02] (*P*=0.001)
NMR cardiovascular risk score (*Z* score)	299	0.08 [0.02 to 0.13] (*P*=0.007)	0.07 [0.01 to 0.13] (*P*=0.017)

Results of mixed models and linear regression analyses assessing the associations between screen time and total cardiometabolic risk, as well as its individual components, markers of insulin resistance, inflammation and atherogenic lipoproteins in the COPSAC2010 and COPSAC2000 cohorts. The table provides both unadjusted and adjusted associations, with the latter controlling for potential confounders (social circumstances, maternal smoking during pregnancy, number of siblings, sedentary time, light activity time, moderate to vigorous activity time, sleep duration and time of sleep onset). In COPSAC2010, we included further adjustments for luteinizing hormone and follicle‐stimulating hormone. The associations are presented as estimates with 95% CIs and corresponding *P* values. Glucose is estimated from HbA1c in COPSAC2010 at age 6 years due to lack of measured glucose at this time point. ApoB indicates apolipoprotein B; COPSAC2000, Copenhagen Prospective Studies on Asthma in Childhood mother–child cohort 2000; COPSAC2010, Copenhagen Prospective Studies on Asthma in Childhood mother–child cohort 2010; CRP, C‐reactive protein; GlycA, glycoprotein acetyls; HbA1c, hemoglobin A1c; HDL, high‐density lipoprotein; HOMA‐IR, Homeostasis Model Assessment of Insulin Resistance; and NMR, nuclear magnetic resonance.

* Triglycerides logged to ensure normalisation.

^†^ Glucose was not available at 6 years in COPSAC2010, and thus substituted with HBA1C for the purposes of mixed modelling.

**Table 3:** Longitudinal Subanalysis of Screen Time From Age 6 to 10 Years With CMR Factors in COPSAC2010

COPSAC2010 outcome variable	Age 6 y [95% Cl] (*P* value)	Screen difference [95% Cl] (*P* value)	Age 10 y adjusted for age 6 y [95% Cl] (*P* value)
Cardiometabolic risk (*Z* score)	0.06 [−0.06 to 0.17] (*P*=0.321)	0.07 [−0.02 to 0.15] (*P*=0.124)	0.11 [0.02 to 0.2] (*P*=0.02)
Waist size, cm	0.29 [−0.39 to 0.97] (*P*=0.397)	0.12 [−0.39 to 0.63] (*P*=0.643)	0.26 [−0.31 to 0.83] (*P*=0.379)
Systolic blood pressure, mm Hg	0.33 [−0.32 to 0.98] (*P*=0.316)	0 [−0.48 to 0.48] (*P*=0.992)	0.14 [−0.4 to 0.68] (*P*=0.615)
HDL cholesterol, mmol/L	0.01 [−0.02 to 0.05] (*P*=0.485)	−0.02 [−0.05 to 0] (*P*=0.068)	−0.03 [−0.05 to 0] (*P*=0.051)
Triglycerides, mmol/L* (very‐low‐density lipoproteinlogged)	0.04 [0 to 0.07] (*P*=0.073)	0.02 [−0.01 to 0.05] (*P*=0.121)	0.04 [0.01 to 0.07] (*P*=0.009)
Glucose, mmol/L	−0.02 [−0.05 to 0.02] (*P*=0.286)	0.02 [−0.01 to 0.04] (*P*=0.18)	0.02 [−0.01 to 0.05] (*P*=0.292)
HbA1c, mmol/mol	−0.05 [−0.32 to 0.21] (*P*=0.688)	0.04 [−0.16 to 0.24] (*P*=0.708)	0.02 [−0.2 to 0.25] (*P*=0.833)
HOMA‐IR	−0.01 [−0.03 to 0.02] (*P*=0.544)	0.03 [0.01 to 0.04] (*P*=0.006)	0.03 [0.01 to 0.05] (*P*=0.006)
High‐sensitivity CRP, mg/L	0.05 [−0.14 to 0.24] (*P*=0.599)	−0.05 [−0.19 to 0.1] (*P*=0.538)	−0.04 [−0.2 to 0.13] (*P*=0.67)
GlycA, mmol/L	0 [−0.01 to 0.01] (*P*=1)	0 [0 to 0.01] (*P*=0.083)	0.01 [0 to 0.01] (*P*=0.049)
ApoB, g/L	0.01 [0 to 0.02] (*P*=0.257)	0 [−0.01 to 0.01] (*P*=0.468)	0 [−0.01 to 0.01] (*P*=0.781)
NMR cardiovascular risk score (*Z* score)	0.01 [−0.09 to 0.11] (*P*=0.885)	0.05 [−0.02 to 0.13] (*P*=0.172)	0.06 [−0.02 to 0.15] (*P*=0.123)

Results of adjusted subanalyses examining the longitudinal associations between screen time at age 6 years, the difference in screen time from age 6 to 10 years, and screen time at age 10 years adjusted for screen time at age 6 years, with defined cardiometabolic risk factors. The associations are presented as estimates with 95% CIs and corresponding *P* values. ApoB indicates apolipoprotein B; CMR, cardiometabolic risk; COPSAC2010, Copenhagen Prospective Studies on Asthma in Childhood mother–child cohort 2010; CRP, C‐reactive protein; GlycA, glycoprotein acetyls; HbA1c, hemoglobin A1c; HDL, high‐density lipoprotein; HOMA‐IR, homeostasis model assessment of insulin resistance; and NMR, nuclear magnetic resonance.

* Triglycerides logged to ensure normalization.

**Figure 1:**
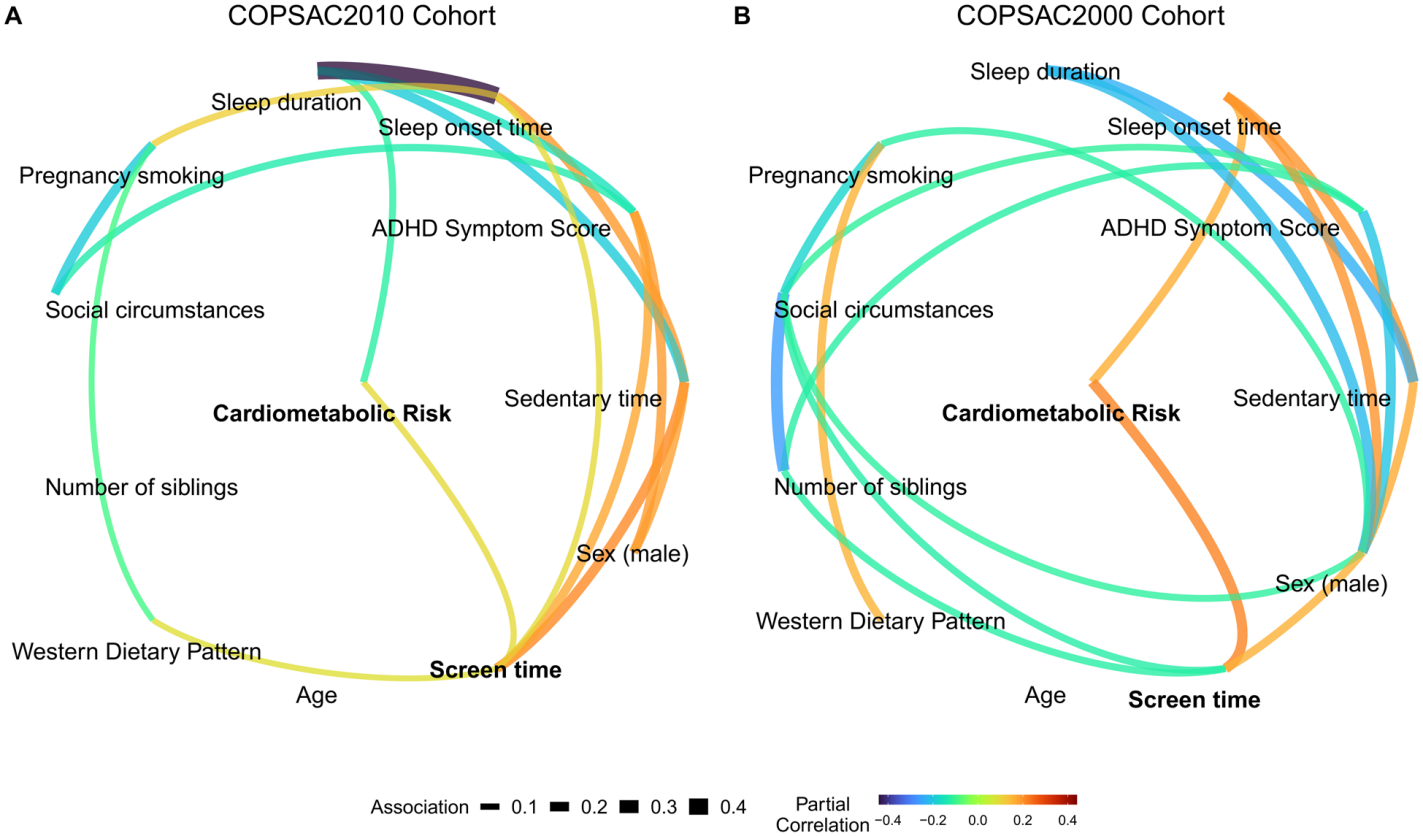
Gaussian graphical models depicting the integrated relationships between cardiometabolic risk, screen time, and other covariates in COPSAC2010 and COPSAC2000. Graphical models illustrate partial correlations (95% CI) among screen time, cardiometabolic risk, and covariates in a multivariable framework. **A**, The COPSAC2010 cohort, showing that screen time and sleep duration (in hours) are directly associated with cardiometabolic risk when accounting for model covariates. **B**, The COPSAC2000 cohort, indicating that both screen time and time of sleep onset have direct associations with cardiometabolic risk. Only statistically significant direct partial associations are shown, accounting for all other included variables. ADHD indicates attention‐deficit/hyperactivity disorder; COPSAC2000, Copenhagen Prospective Studies on Asthma in Childhood mother–child cohort 2000; and COPSAC2010, Copenhagen Prospective Studies on Asthma in Childhood mother–child cohort 2010.

To illustrate the relationship between CMR and covariates in the COPSAC2010 cohort, we used Gaussian graphical models (Figure 1A, sex‐stratified models Figure S5). Figure 1A highlights the significant partial correlations between screen time and CMR in COPSAC2010, even when accounting for model covariates. These models revealed a direct association between sleep duration and CMR, so we assessed if it may act as a potential mediator in the association between screen time and CMR. A mediation analysis indicated that 12.0% (*P*=0.030) of the association between screen time and CMR was mediated through sleep duration.

Using screen time data available from age 6 years, we conducted a series of subanalyses to provide longitudinal insights into the relationship between screen time and CMR factors at age 10 years. An increase per hour of screen time from age 6 to 10 years was associated with measures of insulin resistance, with an increase in Homeostatic Model Assessment for Insulin Resistance (0.03 [0.01–0.04], *P*=0.006). Moreover, screen time at age 10 years, adjusted for screen time at age 6 years, was significantly associated with total CMR (0.11 [0.02–0.2], *P*=0.020) (Table 3). Associations stratified by weekday and weekend screen time can be seen in Table S6.

### Screen Time Is Associated With Cardiometabolic Risk in COPSAC2000

In COPSAC2000 at age 18 years, we likewise identified significant positive associations between screen time and CMR (0.13 [0.07–0.2], *P*<0.001). Furthermore, several CMR factors were significantly associated including waist circumference (1.30 [0.76–1.84], *P*<0.001), SBP (0.63 [0.15–1.11], *P*=0.011), HDL cholesterol (−0.01 [−0.03 to 0], *P*=0.032), glycoprotein acetyls (0.01 [0.01–0.02], *P*<0.001), and apolipoprotein B (0.01 [0.01–0.02], *P*=0.001) (Table 2). Sex‐stratified analysis again revealed stronger associations for boys for CMR (0.14 [0.06–0.22], *P*=0.001), waist circumference (1.56 [0.89–2.22], *P*<0.001), and SBP (0.9 [0.24–1.56], *P*=0.008). However, no statistically significant interaction effects were observed between sexes. Further adjustments for a Western dietary pattern metabolome score, derived from newborn dry blood spots, did not meaningfully change these findings, and similar robustness was observed when additionally adjusting for asthma status by age 18 years in COPSAC2000 (Table S5).

To illustrate the relationship between CMR and covariates in the COPSAC2000 cohort, we used Gaussian graphical models (Figure 1B, sex stratified models Figure S5). Figure 1B highlights the robust association between screen time and total CMR in COPSAC2000, when accounting for model covariates. Sleep onset time also had an independent association with CMR; however, there was no association with screen time, indicating that it does not serve as a mediator in this relationship.

Screen time, whether through phones, televisions, or gaming, consistently demonstrated a positive association with multiple CMR factors (Table S7). Associations stratified by weekday and weekend screen time can be seen in Table S6.

### Screen Time and Body Anthropometry in COPSAC2010 and COPSAC2000

In COPSAC2010, at age 10 years, we found no significant associations between screen time and various body anthropometric measures, including body mass index, body weight, fat mass, fat percent, muscle mass, skeletal mass, bone mass, fat‐free mass, fat mass index, and fat‐free mass index in both girls and boys (Table 4).

**Table 4:** Sex‐Stratified Associations Between Screen Time and Body Anthropometrics in COPSAC2010 and COPSAC2000

Body anthropometrics COPSAC2010	No.	Female adjusted [95% Cl] (*P* value)	No.	Male adjusted [95% Cl] (*P* value)	*P* value interaction
BMI (actual)	269	0.11 [−0.19 to 0.4] (*P*=0.473)	291	0.11 [−0.11 to 0.33] (*P*=0.339)	*P*=0.774
Body weight, kg	270	0.18 [−0.64 to 1.01] (*P*=0.669)	292	0.35 [−0.3 to 1.01] (*P*=0.292)	*P*=0.788
Fat mass, kg	269	0.14 [−0.24 to 0.52] (*P*=0.473)	291	0.22 [−0.09 to 0.52] (*P*=0.164)	*P*=0.537
Fat percent, %	269	0.28 [−0.23 to 0.8] (*P*=0.284)	291	0.3 [−0.14 to 0.75] (*P*=0.182)	*P*=0.517
Muscle mass, kg	269	0.07 [−0.41 to 0.55] (*P*=0.778)	291	0.1 [−0.27 to 0.48] (*P*=0.594)	*P*=0.846
Skeletal mass, kg	269	0.04 [−0.25 to 0.33] (*P*=0.776)	291	0.06 [−0.16 to 0.28] (*P*=0.59)	*P*=0.844
Bone mass, kg	269	0 [−0.02 to 0.03] (*P*=0.741)	291	0 [−0.01 to 0.02] (*P*=0.606)	*P*=0.77
Fat‐free mass, kg	269	0.07 [−0.44 to 0.58] (*P*=0.776)	291	0.11 [−0.29 to 0.5] (*P*=0.594)	*P*=0.843
Fat mass index	269	0.08 [−0.09 to 0.24] (*P*=0.362)	291	0.09 [−0.04 to 0.22] (*P*=0.168)	*P*=0.551
Fat‐free mass index	269	0.03 [−0.12 to 0.19] (*P*=0.672)	291	0.02 [−0.09 to 0.13] (*P*=0.688)	*P*=0.962

Body anthropometrics COPSAC2000	No.	Female adjusted [95% Cl] (*P* value)	No.	Male adjusted [95% Cl] (*P* value)	*P* value interaction
BMI, actual	181	0.33 [−0.02 to 0.67] (*P*=0.065)	173	0.52 [0.28 to 0.76] (*P*<0.001)	*P*=0.328
Body weight, kg	181	0.8 [−0.2 to 1.79] (*P*=0.12)	175	1.72 [0.83 to 2.61] (*P*<0.001)	*P*=0.168
Fat mass, kg	181	0.52 [−0.11 to 1.15] (*P*=0.106)	173	1.07 [0.58 to 1.56] (*P*<0.001)	*P*=0.159
Fat percent, %	181	0.37 [−0.12 to 0.86] (*P*=0.145)	173	0.84 [0.46 to 1.22] (*P*<0.001)	*P*=0.108
Muscle mass, kg	181	0.26 [−0.18 to 0.71] (*P*=0.251)	173	0.64 [0.15 to 1.12] (*P*=0.011)	*P*=0.287
Skeletal mass, kg	181	0.16 [−0.1 to 0.42] (*P*=0.228)	173	0.37 [0.07 to 0.67] (*P*=0.015)	*P*=0.335
Bone mass, kg	181	0.01 [−0.01 to 0.04] (*P*=0.278)	173	0.03 [0.01 to 0.06] (*P*=0.007)	*P*=0.255
Fat‐free mass, kg	181	0.27 [−0.19 to 0.74] (*P*=0.252)	173	0.67 [0.16 to 1.18] (*P*=0.011)	*P*=0.285
Fat mass index	181	0.2 [−0.02 to 0.43] (*P*=0.082)	173	0.33 [0.18 to 0.47] (*P*<0.001)	*P*=0.344
Fat‐free mass index	181	0.12 [−0.02 to 0.27] (*P*=0.091)	173	0.19 [0.07 to 0.31] (*P*=0.002)	*P*=0.397

Results of adjusted sex‐stratified analyses assessing the associations between screen time and various body anthropometric measures in the COPSAC2010 and COPSAC2000 cohorts. The measures include BMI, body weight, fat mass, fat percent, muscle mass, skeletal mass, bone mass, fat‐free mass, fat mass index, and fat‐free mass index. The associations are presented separately for girls and boys in each cohort. The results highlight the significant associations between increased screen time and various body anthropometric measures, with differences noted between girls and boys. The associations are presented as estimates with 95% CIs and corresponding *P* values. BMI indicates body mass index; COPSAC2000, Copenhagen Prospective Studies on Asthma in Childhood mother–child cohort 2000; and COPSAC2010, Copenhagen Prospective Studies on Asthma in Childhood mother–child cohort 2010.

In COPSAC2000 at age 18 years, we found several significant associations between screen time and body anthropometrics, but the associations varied by sex. In girls, screen time was associated with increased body mass index (0.37 [0.02–0.72], *P*=0.039) and had borderline associations with increased body weight and fat mass. In contrast, male screen time was associated with all measured body anthropometrics, including body mass index, body weight, fat mass, fat percentage, muscle mass, skeletal mass, bone mass, fat‐free mass, fat mass index, and fat‐free mass index (*P*≤0.015) (Table 4), but with no significant interaction between sexes (*P*>0.13).

### Screen Time Has a Distinct Blood Metabolome Signature and Associations With CVD Risk

We used a machine learning prediction model in COPSAC2010, using screen time as the classifier and a targeted NMR blood metabolomics platform as the predictor. Our model considered 173 metabolic biomarkers and was regularized to retain only the most influential predictors, resulting in a final model that included 37 screen time‐associated biomarkers (Figure S6). This model, with screen time *Z*‐scored for cross‐data set interpretation trained in COPSAC2010, predicted screen time in both COPSAC2010 (0.80 [0.32–1.28], *P*=0.001), and COPSAC2000 (0.14 [0.03–0.26], *P*=0.014) (adjusted associations). These findings suggest that metabolic disturbances associated with screen time are a robust predictor of screen time across independent cohorts.

Additionally, we assessed the associations between screen time and cardiovascular risk in both COPSAC cohorts using an NMR cardiovascular risk score, based on sex‐stratified Cox proportional hazards models for 10‐year CVD risk trained in the UK Biobank. Screen time showed a positive trend with the NMR CVD score in COPSAC2010 at age 10 years (0.06 [−0.02 to 0.13], *P*=0.15) and was significantly associated in COPSAC2000 at age 18 years (0.07 [0.01–0.13], *P*=0.017) in adjusted models (Table 2).

### Sleep Is a Modifying Factor in the Association Between Screen Time and Cardiometabolic Risk

We explored how different lifestyle factors may influence the relationship between screen time and total CMR, focusing on lifestyle behaviors such as sedentary and light activity time, sleep duration and onset, and dietary patterns. Figure S7 visually represents the associations of these lifestyle factors across quartiles with screen time for COPSAC2010 and COPSAC2000, respectively. In interaction analysis, sedentary time, light activity time, and a Western dietary pattern showed no significant contextual associations with screen time; however, sleep duration and onset emerged as modifiers in the screen time–CMR relationship.

**Figure 2:**
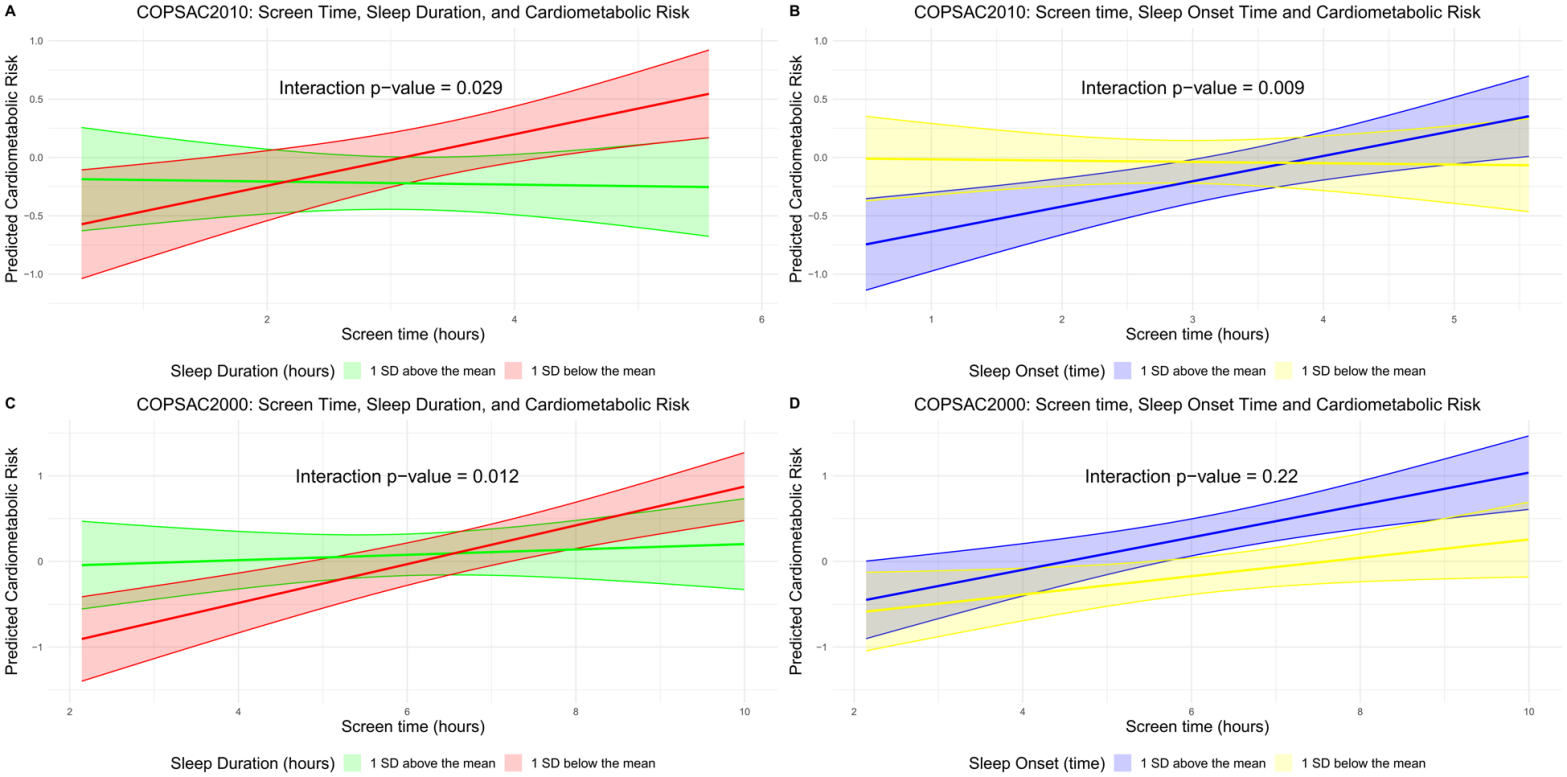
Modulating effects of sleep duration and onset time on the relationship between screen time and cardiometabolic risk in COPSAC2010 and COPSAC2000. Potential modulating effects of sleep duration and onset time on the relationship between screen time and total cardiometabolic risk. **A** and **B**, Relationships for the COPSAC2010 cohort. **C** and **D**, Relationships for the COPSAC2000 cohort. COPSAC2000 indicates Copenhagen Prospective Studies on Asthma in Childhood mother–child cohort 2000; and COPSAC2010, Copenhagen Prospective Studies on Asthma in Childhood mother–child cohort 2010.

In COPSAC2010, there was significant effect moderation between screen time and sleep duration (*P*=0.029), indicating that the positive association of screen time on CMR increased as the amount of sleep decreased (Figure 2A). Similarly, there was significant effect moderation between screen time and sleep onset time (*P*=0.009), suggesting that later sleep onset may exacerbate the detrimental effects of screen time on CMR (Figure 2B).

In COPSAC2000, there was a replication of this finding, with a significant moderation between screen time and sleep duration on total CMR (*P*=0.012) (Figure 2C). This suggests a similar trend as in COPSAC2010, whereby decreased sleep duration may exacerbate the negative impact of screen time on cardiometabolic health. There was a directional but nonsignificant interaction between screen time and sleep onset time in COPSAC2000 (*P*=0.22) (Figure 2D).

We also conducted sex‐stratified analyses to examine potential sex differences in moderating effects. In COPSAC2010 boys, the interaction between screen time and sleep duration was more pronounced (*P*=0.031) (Figure S8A). In contrast, among COPSAC2000 girls, the interaction between screen time and sleep onset time was significant (*P*=0.005), suggesting that the detrimental moderation on CMR of increased screen time is magnified when the sleep onset is later in female adolescents (Figure S8B).

## DISCUSSION

Our study establishes a clear association in 2 independent mother–child cohorts between screen time and elevated CMR in both childhood and adolescence, a relationship that persists even when accounting for objective measures of sleep, diet quality, physical activity, and pubertal development. In childhood, lifestyle factors such as sleep duration and onset were found to significantly moderate the relationship between screen time and CMR, with sleep duration also identified as a potential mediator. In adolescence, time of sleep onset had independent significant associations with CMR, and sleep duration significantly moderated the relationship. We used a machine learning model trained in 1 cohort to uncover a unique metabolic signature associated with screen time, which despite significant cohort characteristic differences was validated in the other cohort, thus suggesting a potential tool for identifying individuals at particular risk of high screen time use. Finally, we used blood metabolome profiles to assess cardiovascular risk using adult‐trained CVD outcome data, finding positive directional associations with screen time in childhood and significant associations in adolescence, perhaps reflecting the later emergence of cumulative metabolic disturbances that are more readily captured by this adult‐derived risk score.

Previous studies have reported no association between self‐reported screen time and CMR in children aged 7 to 12 years.^18^ However, our study indicates that such an association becomes more pronounced in adolescence, suggesting that the associations between screen time and cardiometabolic health may differ across developmental stages. This is supported by a further study, which found that higher screen time in adolescence was associated with higher odds of select indicators of cardiometabolic disease in adulthood, including obesity, hypertension, hyperlipidemia, and diabetes.^6^ Although our observed effect sizes may appear modest, they reflect consistent shifts across multiple cardiometabolic domains per additional hour of screen time, and may indicate early divergence in cardiometabolic health trajectories.

Our study further enriches the literature by identifying sleep duration and time of onset as key modulating factors in the screen time‐CMR association in childhood. We validated the moderating effect of sleep duration on CMR in COPSAC2000 and found that time of sleep onset was a significant independent risk factor in adolescence. However, a recent study of 3000 participants, aged 6 to 17 years, using self‐reported measures of sleep and screen time, found no moderating role of sleep on screen times associated with cardiometabolic risk.^31^ Our findings align with existing literature that link shorter sleep duration with higher CMR in adolescents.^32^ Evening screen has been suggested to disrupt circadian rhythms, delay melatonin production, and shorten sleep duration.^8^ These potential mechanisms may contribute to hormonal dysregulation, increased appetite, and weight gain in children and adolescents.^33^ Interventions focused on limiting screen exposure before bedtime and promoting sleep hygiene practices have been highlighted as promising strategies to reduce screen time's potential metabolic impact in vulnerable youth populations. Although our analysis focused on sleep duration and timing, emerging evidence suggests that sleep quality may also contribute to metabolic health and could be an important target for future interventions. Our findings may suggest actionable targets; for instance, in childhood where sleep onset time and total sleep duration were closely linked, encouraging earlier bedtimes may increase overall sleep time and thus potentially mitigate adverse cardiometabolic factors associated with screen time in childhood.

A unique strength of our study is the use of 2 prospective mother–child cohorts with deep phenotyping and longitudinal follow‐up. The COPSAC2010 cohort, in particular, used repeated outcome and exposure data, allowing for us to account for interindividual variation in participants, thus providing more robust inference. Furthermore, our application of a machine learning approach uncovered a robust metabolic signature of screen time, which was a significant predictor of actual screen time, independent of other potential confounding factors. The identified metabolic signature included 37 biomarkers, many of which have previously been linked to obesity‐related traits and lipid metabolism, such as elevated triglycerides in various very low‐density lipoprotein subclasses and reduced large HDL cholesterol fractions.^34^ These metabolomic features suggest that increased screen time may be associated with early alterations in lipid transport, lipoprotein particle size, and fatty acid balance, providing mechanistic insight into the observed cardiometabolic risk. To the best of our knowledge, we are also the first study to implement CVD risk scores modeled on adult CVD outcomes in independent childhood and implement these for inference. Thus, our comprehensive approach, which accounts for objective lifestyle measures, establishes a robust link between screen time and CMR during both childhood and adolescence. However, our study also has limitations. The observational nature of the study design limits our ability to infer causality, and we cannot rule out residual confounding. Furthermore, our reliance on reported measures for screen time could introduce bias.^13^ However, this methodology is widely accepted in the field,^35^ and the prevalence of high screen time usage is corroborated by studies using wearable cameras to monitor children's screen activities.^36^ Future studies would benefit from more granular and quantitatively assessed measures of screen use, including precise logging of duration, device type, and time‐of‐day exposure, to better capture the diversity and intensity of screen behaviors. These improvements could enhance our understanding of the biological pathways through which screen time may impact cardiometabolic risk. Furthermore, although many of our lifestyle measures, such as sleep and physical activity, were objective, our dietary assessments were self‐reported and therefore potentially subject to recall or social desirability bias. Finally, the nature of our moderation modeling of lifestyle factors and mediation analysis were exploratory and constituted subanalysis. Therefore, although these findings provide valuable context, they should be interpreted with caution and considered as hypothesis‐generating rather than definitive. Although associations appeared stronger in boys, formal interaction tests did not show statistically significant sex differences in the relationship between screen time and cardiometabolic outcomes.

Moving forward, future studies should focus on using objective measures of screen time and related lifestyle behaviors. Our findings suggest that sleep duration and onset may play a significant role in moderating the impact of screen time on cardiometabolic health in childhood. Associations between sleep duration and cardiometabolic risk in childhood and adolescence are well‐documented,^37^ with suggested mechanisms including circadian rhythm misalignment,^38^ sodium retention secondary to insulin resistance,^39^ and increased sympathetic nervous system activity.^37^ Our findings indicate a contextual association between screen time and cardiometabolic risk, which becomes stronger in cases of decreased sleep. This may be explained by the reduction in melatonin levels caused by exposure to screen light in the evening, leading to disturbances in circadian rhythms that exacerbate this relationship.^40^ Moreover, our study suggests that the associations between screen time and cardiometabolic risk are independent of lifestyle behaviors, including sedentary time, suggesting that independent mechanisms whereby screen time may influence cardiometabolic risk, such as poor stress regulation and high sympathetic arousal.^41^

Our findings support the consideration of screen time as an independent behavioral risk factor for early cardiometabolic dysregulation, which could inform future strategies for early risk identification and intervention. Future studies are needed to confirm and further elucidate these relationships. Our results also highlight the potential usefulness of machine learning approaches in identifying key metabolic signatures of screen time. Despite the parental‐/self‐reported nature of our screen time data, our model was able to predict screen time across 2 distinct cohorts with significantly different baseline characteristics, suggesting that the metabolic signature of screen time is robust and consistent across varying populations. These insights could be useful in future research to both identify individuals at risk of high screen time and its associated CMR and inform interventions to reduce screen time and improve cardiometabolic health.

In conclusion, our findings from 2 independent mother–child cohorts emphasize the consistent association between screen time and elevated cardiometabolic risk, with sleep duration and time of onset acting as potential contextual factors. Distinct patterns of associations when juxtaposing these cohorts suggest that interventions aimed at reducing CMR may need to be tailored distinctly for children and adolescents, considering the varying influences of lifestyle factors at these stages. These insights underline the importance of comprehensive, multifaceted strategies to mitigate CMR in childhood and adolescence, with a particular focus on reducing screen time and promoting healthier sleeping habits.

## Sources of Funding

All funding received by COPSAC is listed on www.copsac.com. The Lundbeck Foundation (grant numbers R16‐A1694 and R269‐2017‐5), the Ministry of Health (grant number 903516), Danish Council for Strategic Research (grant number 0603‐00280B), and the Capital Region Research Foundation have provided core support to the COPSAC research center. This project has received funding from the European Research Council under the European Union's Horizon 2020 research and innovation program (grant agreement number 946228) (B.C.). M.A.R. is funded by the Novo Nordisk Foundation (grant number NNF21OC0068517).

## Disclosures

None.

## Supporting information

Data S1
Tables S1–S7
Figures S1–S8
References 42–50
