# Supplementary material for: Screen Time Is Associated With Cardiometabolic and Cardiovascular Disease Risk in Childhood and Adolescence
Source: J Am Heart Assoc. 2025 Aug 6;14(16):e041486. doi: 10.1161/JAHA.125.041486 (PMC12533741; doi:10.1161/JAHA.125.041486)
Supplement: Supplementary file 1 — Data S1 Tables S1–S7 Figures S1–S8 References 42–50 [file JAH3-14-e041486-s001.pdf]

SUPPLEMENTAL MATERIAL

Data S1. Supplemental Methods

Screen time Measurement

The primary exposure under investigation was screen time, which was derived from questionnaire responses in both COPSAC2010 and COPSAC2000 cohorts, focusing on discretionary screen time.

For COPSAC2010, parents reported their children's average screen time at the 6- and 10-year visits, which included average usage of electronic screens and television. The response options included `None`, `Don't know`, `Max ½ hour`, `Max 1`, `Max 2`, `Max 3`, `Max 4` and `Over 4 hours` in hourly increments for weekdays, and for weekends or holidays, `Max 4 hours`, `Max 5 hours`, `Max 6 hours` and `Over 6 hours` were also considered.

For COPSAC2000, during the 18-year visit, participants were similarly asked to detail their screen time usage in hourly increments up to `over 4 hours` Monday to Thursday, and up to `over 6 hours` for Friday to Sunday. The COPSAC2000 questionnaire differentiated three types of screen time: daily time spent watching movies on a TV screen, time spent gaming on a TV screen, and time spent using screens from phones, tablets, or computers during leisure. The total screen time was calculated by summing these three components.

The responses indicating 'Over 4' and 'Over 6' were transformed to five and seven hours respectively, as the subsequent highest values were `Max 4' and 'Max 6' for weekdays and weekends respectively. To derive screen time for each cohort, a weighted average for weekdays and weekends was calculated. This was done by multiplying the weekday hours by 5, the weekend hours by 2, and dividing the sum by 7. For COPSAC2000, however, the questionnaire's unique division of Monday-Thursday and Friday-Sunday screen time necessitated the use of 4 and 3 as the respective weighting factors.

Cardiometabolic Outcomes

A CMR score was calculated if 3 or more of the 5 constituent variables were available, for individuals eligible, but with missing data, missing variables were imputed using the imputePCA function, from the missMDA R package, relative to the available variables (42). In COPSAC2010, for longitudinal

analysis we lacked glucose at 6 years, and thus substituted HBA1C for Glucose in calculating CMR (Estimated Glucose (mmol/L) = (0.0915 × HbA1c [mmol/mol]) + 2.15).

SBP was measured by trained research assistants during clinical visits: Three measures were taken consecutively, resting in a sitting position, and the average of the second and third measurements was used in our analysis. Blood samples were taken from the children at age 6 and 10 years in COPSAC2010, and at 18 years in COPSAC2000. Samples were sent to the on-site hospital biochemistry department, and processed on the same day. Blood samples were taken fasting, with the exception of the 6 year visit in COPSAC2010. As triglycerides are sensitive to postprandial variation (43), we removed 26 childrens triglycerides with unrealistically high measurements (>2 mmol/l) at the 6 year visit. Further we log-transformed triglycerides in both cohorts due to a right-skewed distribution. HOMA-IR was calculated using insulin and glucose in COPSAC2000 (44) and using C-peptide and glucose in COPSAC2010 (45).

Body Anthropometrics

Waist circumference was measured with a tape, using the navel as a fixed point and taking the mean of two measures during inspiration and expiration. Height was measured with a stadiometer, which was calibrated yearly, and weight was measured without clothes using calibrated digital scales.

Covariates

Specifically, we adjusted for the first three principal components of the nutrient estimates from a food frequency questionnaire encompassing 145 distinct food items, designed to capture the consistent dietary habits of children, further details can be found in existing work (46). In COPSAC2010, ADHD symptom scores were derived from the total score of the ADHD Rating Scale questionnaire, which was completed by parents at the 10-year clinical visit (47). In COPSAC2000, participants were asked to complete the Adult ADHD Self-Report Scale v1.1 at the 18-year clinical cohort visit (48). Missing covariate data for multivariable models were imputed, relative to other model covariates, using the missMDA R package (42) using the imputePCA function, thus participants were only excluded from our main analysis when missing exposure or outcome data.

Statistical Analysis

We carried out a series of sensitivity analyses in both cohorts to evaluate the impact of screen time after additional adjustments. In COPSAC2010, we further adjusted for the results of two prenatal randomised controlled trials of n3-LCPUFA supplementation and high-dose vitamin D during pregnancy (14). In COPSAC2000, we further adjusted for a newborn dry blood spot metabolome-derived Western dietary pattern score. This metabolome score is detailed in previous research (46). We opted not to include the metabolome derived dietary pattern in our main analysis in COPSAC2000, as it may not accurately reflect the participants dietary pattern, but may capture some of the variance explained by diet, as parental eating behaviours influence children's food choices (49).

We used gaussian graphical network models via the framework described by Williams and Rast to interpret the results (50). We opted to exclude light activity and MVPA time from graphical models, to avoid problems with multicollinearity when interpreting graphical models, and these were not univariately associated with total CMR ($p>0.1$). In models assessing independent associations of sleep and physical activity on CMR, we removed other accelerometer-derived variables to avoid collinearity. We further opted to exclude LH/FSH to ease comparison of associations between cohorts.

Missing data for Nightingale (0.45% in COPSAC2010, 0.06% in COPSAC2000) used in this modelling was imputed using the missMDA R package (v1.18) (42). In order to ensure robust extrapolation and minimise the risk of overfitting, we opted to use a less complex sPLS model for external validation in COPSAC2000, restricting the model to 1 component.

Cohort Baseline Characteristics	COPSAC2010 Cohort	COPSAC2000 Cohort	p-value
n =	630	364	
Age (years) (mean (SD))	10.30 (0.39)	17.73 (0.57)	<0.001
Male Sex (%)	324 (51.4)	179 (49.2)	0.536
Caucasian Race (%)	603 (95.7)	352 (96.7)	0.546
Income type (%)			<0.001
Low Income	51 (8.1)	98 (27.9)	
Medium Income	334 (53.0)	224 (63.8)	
High Income	245 (38.9)	29 (8.3)	
Maternal Education Level at Birth			<0.001
Low level educational attainment	46 (7.3)	198 (56.7)	
Medium level educational attainment	390 (61.9)	96 (27.5)	
High level educational attainment	194 (30.8)	55 (15.8)	
Maternal age at birth (years) (mean (SD))	32.45 (4.30)	30.06 (4.59)	<0.001
Birthweight (kilograms) (mean (SD))	3.54 (0.55)	3.53 (0.53)	0.726
Gestational age (days) (mean (SD))	279.13 (11.69)	282.34 (10.81)	<0.001
Cesarean section (%)	134 (21.3)	78 (21.4)	1
Maternal smoking during pregnancy (%)	45 (7.1)	83 (22.8)	<0.001
Siblings (mean (SD))	1.47 (0.94)	1.24 (0.91)	<0.001

Table S1. Baseline Characteristics of COPSAC2010 and COPSAC2000. Baseline characteristics of the COPSAC2010 and COPSAC2000 cohorts. The characteristics include age, sex, race, income type, maternal education level at birth, maternal age at birth, birthweight, gestational age, caesarean section, maternal smoking during pregnancy, and number of siblings. Low, medium, and high income are defined as <50,000, 50,000 - 110,000, and >110,000 euro respectively. Low, medium and high level educational attainment are defined as “primary, secondary, or college graduate”, “tradesman or bachelor's degree” and “Masters degree”, respectively. The table provides a comprehensive overview of the demographic and clinical characteristics of the cohorts, highlighting significant differences between the two cohorts in several characteristics.

Screentime Covariate Associations COPSAC2010		Bottom Tertile	Middle Tertile	Top Tertile	p-value
	n=	260	186	184	-
Age (years) (mean (SD))	630	10.24 (0.33)	10.33 (0.40)	10.35 (0.44)	0.006
Number of Siblings (mean (SD))	630	1.40 (0.92)	1.45 (0.85)	1.59 (1.04)	0.11
Male Sex (%)	630	106 (40.8)	101 (54.3)	117 (63.6)	<0.001
Maternal Pregnancy Smoking (%)	629	132 (50.8)	101 (54.3)	87 (47.5)	0.43
Social circumstances (mean (SD))	630	0.08 (0.98)	0.10 (1.05)	-0.09 (0.94)	0.103
Sedentary time (hours) (mean (SD)	480	8.16 (1.03)	8.39 (1.24)	9.01 (1.24)	<0.001
Light activity time (hours) (mean (SD))	480	3.97 (0.60)	3.82 (0.73)	3.67 (0.63)	<0.001
Moderate Activity time (hours) (mean (SD))	480	2.21 (0.48)	2.20 (0.56)	1.98 (0.53)	<0.001
Vigorous activity time (hours) (mean(SD))	480	0.42 (0.22)	0.45 (0.23)	0.39 (0.18)	0.066
Sleep (hours) (mean (SD))	480	9.20 (0.60)	9.01 (0.81)	8.84 (0.85)	<0.001
Sleep Onset Time (hours) (mean (SD))	480	21.70 (0.80)	21.85 (0.76)	22.05 (0.86)	<0.001
LH (Puberty) (mean (SD))	505	0.42 (0.64)	0.58 (0.92)	0.57 (1.01)	0.113
FSH (Puberty) (mean (SD))	471	3.05 (2.03)	3.14 (2.04)	2.62 (1.81)	0.062
ADHD Symptom Score (mean (SD))	586	7.85 (6.97)	9.65 (8.33)	12.92 (10.15)	<0.001
Varied dietary pattern (PC1) (mean (SD))	604	0.04 (1.00)	0.09 (0.93)	-0.15 (1.06)	0.057
Western dietary pattern (PC2) (mean (SD))	604	-0.09 (0.97)	-0.07 (0.85)	0.20 (1.15)	0.006
High Dietary Fats dietary pattern (PC3) (mean (SD))	604	-0.09 (0.94)	-0.03 (1.07)	0.12 (0.99)	0.095
Fish oil prenatal RCT (%)	629	132 (50.8)	101 (54.3)	87 (47.5)	0.43
High dose Vitamin D prenatal RCT (%)	630	115 (52.8)	83 (53.9)	67 (43.2)	0.109
**Screentime Covariate Associations COPSAC2000**		**Bottom Tertile**	**Middle Tertile**	**Top Tertile**	**p-value**
	n=	122	121	121	-
Age (years) (mean (SD))	361	17.71 (0.52)	17.77 (0.56)	17.71 (0.62)	0.672
Number of Siblings (mean (SD))	335	1.29 (0.91)	1.24 (0.90)	1.19 (0.92)	0.732
Male Sex (%)	364	51 (41.8)	52 (43.0)	76 (62.8)	0.001
Maternal Pregnancy Smoking (%)	364	18 (14.8)	32 (26.4)	33 (27.3)	0.034
Social circumstances (mean (SD))	349	0.24 (1.07)	-0.08 (0.93)	-0.12 (0.98)	0.012
Sedentary time (hours) (mean (SD)	281	10.69 (1.22)	10.98 (1.35)	11.04 (1.34)	0.143
Light activity time (hours) (mean (SD))	281	3.59 (0.70)	3.57 (0.79)	3.41 (0.74)	0.2
Moderate Activity time (hours) (mean (SD))	281	1.61 (0.50)	1.55 (0.54)	1.60 (0.61)	0.796
Vigorous activity time (hours) (mean(SD))	281	0.08 (0.06)	0.07 (0.08)	0.10 (0.13)	0.124
Sleep (hours) (mean (SD))	281	7.93 (0.78)	7.76 (0.82)	7.52 (0.93)	0.005
Sleep Onset Time (hours) (mean (SD))	281	24.38 (1.19)	24.50 (1.08)	24.49 (1.29)	0.754
ADHD Symptom Score (mean (SD))	344	3.85 (3.59)	4.21 (4.04)	4.07 (4.04)	0.783
Western Dietary Pattern Metabolome Score in DSB (SD) (mean (SD))	343	-0.09 (0.98)	-0.10 (0.95)	0.12 (0.99)	0.157

Table S2. Screen Time Covariate Associations in COPSAC2010 and COPSAC2000. Associations between screen time and various covariates in the COPSAC2010 and COPSAC2000 cohorts. In the COPSAC2010 cohort, the covariates are stratified by screen time tertiles (<2.85 hours, ≥2.85 hours - <3.86 hours, ≥3.86 hours), while in the COPSAC2000 cohort, the covariates are stratified by screen time tertiles (≤5 hours, >5 hours - <6.43 hours, ≥6.43 hours). The table provides a comprehensive overview of the associations between screen time and these covariates, highlighting significant associations in both cohorts. In addition to the stratified data, the table also provides univariate linear estimates and inference for each covariate in the right two columns, offering a more detailed understanding of the relationship between screen time and each covariate.

Descriptive Sex Analysis of COPSAC2010	Female	Male	p-value	Descriptive Sex Analysis of COPSAC2000	Female	Male	p-value
n =	306	324	-	n=	185	179	-
6 years Average Screen Time (hours) (mean (SD))	1.96 (0.91)	2.04 (0.89)	0.275	-	-	-	-
10 years Average Screen Time (hours) (mean (SD))	2.97 (1.16)	3.38 (1.23)	<0.001	18 years Average Screen Time (hours) (mean (SD))	5.67 (1.75)	6.55 (2.35)	<0.001
6 years Cardiometabolic Risk (Z-score)	-0.02 (1.08)	-0.01 (0.94)	0.988	-	-	-	-
6 years Waist size (cm)	55.21 (3.85)	55.05 (3.39)	0.58	-	-	-	-
6 years Systolic Blood Pressure (mmHg)	100.56 (6.08)	101.61 (6.27)	0.04	-	-	-	-
6 years HDL Cholesterol (mmol/l)	1.42 (0.34)	1.45 (0.32)	0.414	-	-	-	-
6 years Triglycerides (mmol/l)	0.93 (0.36)	0.91 (0.38)	0.598	-	-	-	-
6 years HBA1C (mmol/mol)	5.43 (0.69)	5.41 (0.44)	0.696	-	-	-	-
10 years Cardiometabolic Risk (Z-score)	-0.04 (1.11)	-0.02 (1.12)	0.861	18 years Cardiometabolic Risk (Z-score)	0.00 (1.17)	0.01 (1.30)	0.946
10 years Waist Size (cm) (mean (SD))	64.48 (7.64)	65.00 (7.20)	0.391	18 years Waist Size (cm) (mean (SD))	79.65 (11.37)	81.36 (11.27)	0.152
10 years Systolic Blood Pressure (mean (SD))	103.83 (6.81)	104.04 (7.05)	0.713	18 years Systolic Blood Pressure (mean (SD))	112.54 (8.91)	119.12 (9.96)	<0.001
10 years HDL mmol/l (mean (SD))	1.53 (0.33)	1.59 (0.31)	0.042	18 years HDL mmol/l (mean (SD))	1.28 (0.27)	1.15 (0.24)	<0.001
10 years Triglyceride mmol/l (mean (SD))	0.76 (0.30)	0.72 (0.34)	0.167	18 years Triglyceride mmol/l (mean (SD))	0.95 (0.42)	0.99 (0.47)	0.419
10 years Glucose mean (SD))	5.49 (0.40)	5.49 (0.39)	0.995	18 years Glucose (mmol/l)	4.99 (0.37)	5.21 (0.40)	<0.001
10 years HB1AC (mmol/mol)	32.10 (2.71)	32.11 (2.67)	0.974	18 years HB1AC (mmol/mol)	31.08 (2.99)	31.64 (2.56)	0.069
10 years HOMA-IR	1.97 (0.22)	1.96 (0.28)	0.593	18 years HOMA-IR	2.86 (1.66)	2.69 (2.15)	0.423
10 years High sensitivity CRP (mg/L)	0.72 (1.98)	0.44 (0.99)	0.106	18 years High sensitivity CRP (mg/L)	1.82 (2.22)	1.60 (2.83)	0.421
10 years GlycA (mmol/l)	0.69 (0.08)	0.67 (0.07)	0.014	18 years GlycA (mmol/l)	0.78 (0.11)	0.74 (0.10)	0.003
10 years ApoB (g/l)	0.71 (0.12)	0.68 (0.11)	0.011	18 years ApoB (g/l)	0.71 (0.16)	0.65 (0.13)	0.002
Age (years) (mean (SD))	10.31 (0.40)	10.28 (0.37)	0.454	Age (years) (mean (SD))	17.71 (0.55)	17.75 (0.58)	0.425
Number of Siblings (mean (SD))	1.44 (0.96)	1.50 (0.92)	0.431	Number of Siblings (mean (SD))	1.25 (0.93)	1.22 (0.81)	0.746
Social circumstances (mean (SD))	0.05 (0.99)	0.03 (1.00)	0.79	Social circumstances (mean (SD))	0.10 (1.03)	-0.08 (0.93)	0.08
Maternal Pregnancy Smoking (%)	22 (7.2)	23 (7.1)	1	Maternal Pregnancy Smoking (%)	46 (24.9)	37 (20.7)	0.407
LH (Puberty) (mean (SD))	0.66 (1.12)	0.37 (0.45)	<0.001	-	-	-	-
FSH (Puberty) (mean (SD))	3.97 (2.17)	2.06 (1.24)	<0.001	-	-	-	-
Sedentary time (hours) (mean (SD)	8.19 (1.18)	8.73 (1.17)	<0.001	Sedentary time (hours) (mean (SD)	10.54 (1.20)	11.33 (1.31)	<0.001
Light activity time (hours) (mean (SD))	4.08 (0.68)	3.61 (0.55)	<0.001	Light activity time (hours) (mean (SD))	3.66 (0.71)	3.37 (0.75)	0.001
Moderate Activity time (hours) (mean (SD))	2.16 (0.53)	2.13 (0.54)	0.516	Moderate Activity time (hours) (mean (SD))	1.61 (0.50)	1.55 (0.60)	0.382
Vigorous activity time (hours) (mean(SD))	0.35 (0.16)	0.49 (0.23)	<0.001	Vigorous activity time (hours) (mean(SD))	0.08 (0.08)	0.10 (0.12)	0.112

Sleep (mean (SD))	9.16 (0.70)	8.93 (0.80)	0.001	Sleep (mean (SD))	8.00 (0.80)	7.43 (0.82)	<0.001
Sleep Onset Time (hours) (mean (SD))	21.81 (0.77)	21.88 (0.86)	0.317	Sleep Onset Time (hours) (mean (SD))	24.15 (1.12)	24.82 (1.17)	<0.001
Number of valid days (Accelerometer)	12.41 (1.40)	12.24 (1.87)	0.261	Number of valid days (Accelerometer)	11.76 (2.09)	11.62 (2.19)	0.588
Varied dietary pattern (PC1) (mean (SD))	0.01 (0.89)	-0.01 (1.10)	0.829	-	-	-	-
Western dietary pattern (mean (SD))	0.04 (0.97)	-0.03 (1.03)	0.393	-	-	-	-
High Dietary Fats dietary pattern PC3 (mean (SD))	-0.01 (0.96)	0.00 (1.03)	0.913	-	-	-	-
-	-	-	-	Western Dietary Pattern (SD) (mean (SD))	-0.04 (0.98)	-0.01 (0.97)	0.833
ADHD Symptom Score (ADHD-RS) (mean (SD))	7.74 (7.25)	11.71 (9.31)	<0.001	ADHD Symptom Score (ASRS) (mean (SD))	4.78 (4.23)	3.28 (3.36)	<0.001
Fish oil prenatal RCT (%)	163 (53.3)	157 (48.6)	0.276	-	-	-	-
High dose Vitamin D prenatal RCT (mean (SD))	122 (48.0)	143 (52.4)	0.362	-	-	-	-
BMI (actual)	17.14 (2.55)	17.02 (2.21)	0.541	BMI (actual)	23.10 (4.27)	22.71 (3.92)	0.371
Body Weight (kilograms)	36.01 (7.35)	35.54 (6.63)	0.426	Body Weight (kilograms)	65.32 (12.23)	75.12 (14.13)	<0.001
Fat Mass (kilograms)	8.65 (3.33)	7.39 (3.04)	<0.001	Fat Mass (kilograms)	19.61 (7.76)	14.20 (7.91)	<0.001
Fat percent (%)	23.38 (4.53)	20.16 (4.48)	<0.001	Fat percent (%)	29.15 (5.99)	18.01 (6.11)	<0.001
Muscle Mass (kilograms)	25.95 (4.33)	26.64 (3.86)	0.046	Muscle Mass (kilograms)	43.38 (5.36)	57.88 (7.43)	<0.001
Skeletal Mass (kilograms)	15.49 (2.57)	15.95 (2.29)	0.026	Skeletal Mass (kilograms)	25.91 (3.15)	34.81 (4.58)	<0.001
Bone Mass (kilograms)	1.42 (0.23)	1.54 (0.19)	<0.001	Bone Mass (kilograms)	2.33 (0.28)	3.04 (0.36)	<0.001
Fat Free Mass (kilograms)	27.37 (4.55)	28.18 (4.04)	0.027	Fat Free Mass (kilograms)	45.70 (5.64)	60.92 (7.78)	<0.001
Fat Mass Index (FMI)	4.11 (1.42)	3.52 (1.28)	<0.001	Fat Mass Index (FMI)	6.95 (2.82)	4.30 (2.37)	<0.001
Fat-free Mass Index (FFMI)	13.04 (1.32)	13.50 (1.08)	<0.001	Fat-free Mass Index (FFMI)	16.17 (1.75)	18.44 (1.87)	<0.001

Table S3. Exposure, Outcome and Covariate Data, Sex-Stratified, for COPSAC2010 and COPSAC2000. Detailed sex-stratified descriptive analysis of the COPSAC2010 and COPSAC2000 cohorts. It includes data on screen time, cardiometabolic risk factors, anthropometrics, and lifestyle behaviours such as physical activity and sleep patterns, stratified by sex. The table provides a comprehensive overview of the sex-related differences in these measures, highlighting significant differences between males and females in both cohorts.

COPSAC2010 6 & 10 year Mixed Model	n=	Girls Adjusted [95% CI] p-value	n=	Boys Adjusted [95% CI] p-value	P-Interaction
Cardiometabolic Risk (Z-score)	240/240	0.02 [-0.08 - 0.12] (p = 0.694)	273/270	0.1 [0.02 - 0.19] (p = 0.013)	p = 0.261
Waist size (cm)	303/289	-0.02 [-0.5 - 0.46] (p = 0.923)	318/311	0.29 [-0.1 - 0.68] (p = 0.145)	p = 0.061
Systolic Blood Pressure (mmHg)	295/288	-0.15 [-0.68 - 0.38] (p = 0.582)	310/307	0.27 [-0.24 - 0.78] (p = 0.303)	p = 0.835
HDL Cholesterol (mmol/l)	228/237	-0.02 [-0.05 - 0.01] (p = 0.117)	263/267	-0.01 [-0.04 - 0.01] (p = 0.21)	p = 0.287
Triglycerides (mmol/l)** Logged	214/237	0.02 [-0.02 - 0.05] (p = 0.399)	251/268	0.04 [0.01 - 0.08] (p = 0.014)	p = 0.771
Estimated Glucose (mmol/l) ++	235/240	0 [-0.06 - 0.05] (p = 0.854)	272/269	0 [-0.03 - 0.03] (p = 0.989)	p = 0.534
**COPSAC2010 10 year Linear Model**	**n=**	**Girls Adjusted [95% CI] p-value**	**n=**	**Boys Adjusted [95% CI] p-value**	**P-Interaction**
Cardiometabolic Risk (Z-score)	249	0.03 [-0.11 - 0.17] (p = 0.708)	276	0.16 [0.05 - 0.27] (p = 0.007)	p = 0.273
Waist size (cm)	289	0.06 [-0.78 - 0.9] (p = 0.891)	311	0.44 [-0.25 - 1.13] (p = 0.211)	p = 0.349
Systolic Blood Pressure (mmHg)	288	-0.31 [-1.07 - 0.45] (p = 0.42)	307	0.57 [-0.12 - 1.27] (p = 0.108)	p = 0.121
HDL Cholesterol (mmol/l)	237	-0.03 [-0.08 - 0.01] (p = 0.133)	267	-0.01 [-0.05 - 0.02] (p = 0.444)	p = 0.484
Triglycerides (mmol/l)** Logged	237	0.02 [-0.02 - 0.07] (p = 0.35)	268	0.06 [0.02 - 0.1] (p = 0.006)	p = 0.346
Glucose (mmol/l)	249	0.01 [-0.04 - 0.05] (p = 0.827)	275	0.01 [-0.02 - 0.05] (p = 0.463)	p = 0.891
HB1AC (mmol/mol)	240	0.01 [-0.32 - 0.35] (p = 0.951)	269	0 [-0.27 - 0.27] (p = 0.991)	p = 0.909
HOMA-IR	235	0.02 [-0.01 - 0.04] (p = 0.206)	264	0.02 [-0.01 - 0.05] (p = 0.151)	p = 0.775
High sensitivity CRP (mg/L)	160	-0.05 [-0.36 - 0.25] (p = 0.724)	178	-0.01 [-0.14 - 0.12] (p = 0.828)	p = 0.539
GlycA (mmol/l)	249	0 [-0.01 - 0.01] (p = 0.408)	275	0.01 [0 - 0.01] (p = 0.064)	p = 0.628
ApoB (g/l)	249	-0.01 [-0.02 - 0.01] (p = 0.209)	275	0.01 [0 - 0.02] (p = 0.184)	p = 0.057
NMR Cardiovascular Risk Score (Z-score)	248	0.1 [-0.02 - 0.22] (p = 0.108)	272	0.02 [-0.09 - 0.12] (p = 0.746)	p = 0.265
**COPSAC2000 18 year Linear Model**	**n=**	**Girls Adjusted [95% CI] p-value**	**n=**	**Boys Adjusted [95% CI] p-value**	**P-Interaction**
Cardiometabolic Risk (Z-score)	168	0.11 [0.01 - 0.21] (p = 0.037)	167	0.14 [0.06 - 0.22] (p = 0.001)	p = 0.497
Waist size (cm)	182	1.01 [0.09 - 1.93] (p = 0.034)	178	1.56 [0.89 - 2.22] (p <0.001)	p = 0.293
Systolic Blood Pressure (mmHg)	182	0.28 [-0.48 - 1.05] (p = 0.471)	177	0.9 [0.24 - 1.56] (p = 0.008)	p = 0.277

HDL Cholesterol (mmol/l)	168	-0.01 [-0.04 - 0.01] (p = 0.23)	167	-0.01 [-0.03 - 0.01] (p = 0.194)	p = 0.980
Triglycerides (mmol/l) **Logged	168	0.03 [0 - 0.07] (p = 0.091)	167	0.01 [-0.02 - 0.04] (p = 0.422)	p = 0.309
Glucose (mmol/l)	168	0 [-0.03 - 0.03] (p = 0.911)	167	0.01 [-0.02 - 0.04] (p = 0.545)	p = 0.544
HB1AC (mmol/mol)	167	-0.23 [-0.48 - 0.03] (p = 0.087)	166	-0.01 [-0.18 - 0.17] (p = 0.951)	p = 0.178
HOMA-IR	165	0.04 [-0.11 - 0.19] (p = 0.611)	165	0.08 [-0.06 - 0.23] (p = 0.25)	p = 0.646
High sensitivity CRP (mg/L)	160	-0.02 [-0.22 - 0.19] (p = 0.855)	167	0.05 [-0.14 - 0.24] (p = 0.614)	p = 0.970
GlycA (mmol/l)	155	0.02 [0.01 - 0.03] (p = 0.001)	144	0.01 [0 - 0.02] (p = 0.006)	p = 0.409
ApoB (g/l)	155	0.02 [0.01 - 0.04] (p = 0.001)	144	0.01 [0 - 0.02] (p = 0.096)	p = 0.109
NMR Cardiovascular Risk Score (Z-score)	155	0.07 [-0.03 - 0.16] (p = 0.17)	144	0.07 [0 - 0.15] (p = 0.056)	p = 0.739

Table S4. Adjusted Analysis, Sex-Stratified, of COPSAC2010 and COPSAC2000. Results of sex-stratified adjusted mixed models and linear regression analyses assessing the associations between screen time and total CMR, as well as its individual components, markers of insulin resistance, inflammation and atherogenic lipoproteins in the COPSAC2010 and COPSAC2000 cohorts. The table provides a comprehensive overview of the sex-related differences in these measures, highlighting significant differences between males and females in both cohorts, including interaction analysis. The associations are presented as estimates with 95% confidence intervals and corresponding p-values.

COPSAC2010/COPSAC2000 Outcome Variables	COPSAC2010 Prenatal RCT Interventions [95% CI] p-value	COPSAC2000 Western Diet Score DBS [95% CI] p-value	COPSAC2000 Asthma Adjusted [95% CI] p-value
Cardiometabolic Risk (Z-score)	0.11 [0.02 - 0.2] (p = 0.013)	0.07 [0.02 - 0.12] (p = 0.004)	0.13 [0.07 - 0.19) (p < 0.001)
Waist size (cm)	0.3 [-0.24 - 0.84] (p = 0.282)	0.77 [0.38 - 1.17] (p < 0.001)	1.29 [0.74 - 1.83] (p < 0.001)
Systolic Blood Pressure (mmHg)	0.19 [-0.32 - 0.7] (p = 0.455)	0.41 [0.06 - 0.76] (p = 0.023)	0.61 [0.13 - 1.09] (p = 0.014)
HDL Cholesterol (mmol/l)	-0.02 [-0.05 - 0] (p = 0.082)	-0.01 [-0.02 - 0] (p = 0.238)	-0.02 [-0.03 - 0] (p = 0.032)
Triglycerides (mmol/l)** Logged	0.04 [0.01 - 0.07] (p = 0.005)	0.01 [-0.01 - 0.02] (p = 0.514)	0.02 [0 - 0.04] (p = 0.119)
Glucose (mmol/l)	0.01 [-0.02 - 0.04] (p = 0.44)	0 [-0.01 - 0.02] (p = 0.763)	0 [-0.02 - 0.03] (p = 0.653)
HB1AC (mmol/mol)	0 [-0.22 - 0.21] (p = 0.966)	-0.03 [-0.13 - 0.08] (p = 0.624)	-0.09 [-0.24 - 0.05] (p = 0.217)
HOMA-IR	0.02 [0 - 0.04] (p = 0.025)	0.06 [-0.01 - 0.13] (p = 0.11)	0.08 [-0.03 - 0.18] (p = 0.143)
High sensitivity CRP (mg/L)	-0.02 [-0.17 - 0.13] (p = 0.798)	0.05 [-0.05 - 0.15] (p = 0.362)	0.01 [-0.13 - 0.15] (p = 0.869)
GlycA (mmol/l)	0 [0 - 0.01] (p = 0.11)	0.01 [0 - 0.01] (p = 0.001)	0.01 [0.01 - 0.02] (p < 0.001)
ApoB (g/l)	0 [-0.01 - 0.01] (p = 0.878)	0.01 [0 - 0.01] (p = 0.024)	0.01 [0.01 - 0.02] (p = 0.001)
NMR Cardiovascular Risk Score (Z-score)	0.05 [-0.03 - 0.12] (p = 0.245)	0.04 [-0.01 - 0.08] (p = 0.111)	0.07 [0.01 - 0.13] (p = 0.017)

Table S5. Adjusted Sub-analyses for ADHD Symptom Loads, Western Dietary Pattern Metabolome Scores, Prenatal RCT Interventions, and Asthma Diagnosis in COPSAC2010 and COPSAC2000. Results of adjusted sub-analyses examining associations between screen time and cardiometabolic risk (CMR) factors. Models are adjusted for: (1) prenatal RCT interventions in COPSAC2010, (2) a Western dietary pattern metabolome score derived from dried blood spots at birth in COPSAC2000, and (3) adolescent asthma diagnosis by age 18 years in COPSAC2000. Associations are presented as effect estimates with 95% confidence intervals and corresponding p-values.

COPSAC2010 10 year Linear Model	Weekdays Adjusted [95% CI] p-value	Weekends Adjusted [95% CI] p-value
Cardiometabolic Risk (Z-score)	0.12 [0.03 - 0.21] (p = 0.009)	0.06 [0 - 0.12] (p = 0.066)
Waist size (cm)	0.39 [-0.16 - 0.94] (p = 0.167)	0.14 [-0.25 - 0.53] (p = 0.472)
Systolic Blood Pressure (mmHg)	0.27 [-0.25 - 0.79] (p = 0.311)	0.02 [-0.35 - 0.38] (p = 0.931)
HDL Cholesterol (mmol/l)	-0.01 [-0.04 - 0.01] (p = 0.281)	-0.02 [-0.04 - 0] (p = 0.017)
Triglycerides (mmol/l)** Logged	0.05 [0.02 - 0.08] (p = 0.001)	0.02 [0 - 0.04] (p = 0.123)
Glucose (mmol/l)	0.01 [-0.02 - 0.04] (p = 0.539)	0.01 [-0.01 - 0.03] (p = 0.448)
HB1AC (mmol/mol)	0.05 [-0.16 - 0.26] (p = 0.64)	-0.06 [-0.21 - 0.09] (p = 0.434)
HOMA-IR	0.02 [0 - 0.04] (p = 0.024)	0.01 [0 - 0.03] (p = 0.083)
High sensitivity CRP (mg/L)	-0.01 [-0.16 - 0.15] (p = 0.905)	-0.02 [-0.13 - 0.09] (p = 0.729)
GlycA (mmol/l)	0.01 [0 - 0.01] (p = 0.047)	0 [0 - 0.01] (p = 0.169)
ApoB (g/l)	0 [-0.01 - 0.01] (p = 0.796)	0 [-0.01 - 0.01] (p = 0.738)
NMR Cardiovascular Risk Score (Z-score)	0.05 [-0.03 - 0.13] (p = 0.242)	0.05 [-0.01 - 0.1] (p = 0.112)
**COPSAC2000 18 year Linear Model**	**Weekdays Adjusted [95% CI] p-value**	**Weekends Adjusted [95% CI] p-value**
Cardiometabolic Risk (Z-score)	0.12 [0.05 - 0.19] (p = 0.001)	0.07 [0.02 - 0.12] (p = 0.004)
Waist size (cm)	1.09 [0.5 - 1.68] (p <0.001)	0.77 [0.38 - 1.17] (p <0.001)
Systolic Blood Pressure (mmHg)	0.51 [-0.01 - 1.03] (p = 0.056)	0.41 [0.06 - 0.76] (p = 0.023)
HDL Cholesterol (mmol/l)	-0.02 [-0.03 - 0] (p = 0.028)	-0.01 [-0.02 - 0] (p = 0.238)
Triglycerides (mmol/l)** Logged	0.03 [0 - 0.05] (p = 0.037)	0.01 [-0.01 - 0.02] (p = 0.514)
Glucose (mmol/l)	0 [-0.03 - 0.02] (p = 0.716)	0 [-0.01 - 0.02] (p = 0.763)
HB1AC (mmol/mol)	-0.16 [-0.32 - 0] (p = 0.055)	-0.03 [-0.13 - 0.08] (p = 0.624)
HOMA-IR	0.08 [-0.03 - 0.19] (p = 0.173)	0.06 [-0.01 - 0.13] (p = 0.11)
High sensitivity CRP (mg/L)	-0.02 [-0.17 - 0.13] (p = 0.769)	0.05 [-0.05 - 0.15] (p = 0.362)
GlycA (mmol/l)	0.01 [0.01 - 0.02] (p <0.001)	0.01 [0 - 0.01] (p = 0.001)
ApoB (g/l)	0.02 [0.01 - 0.03] (p <0.001)	0.01 [0 - 0.01] (p = 0.024)
NMR Cardiovascular Risk Score (Z-score)	0.09 [0.03 - 0.16] (p = 0.004)	0.04 [-0.01 - 0.08] (p = 0.111)

Table S6. Weekday and Weekend Sub-analysis of Screen Time with CMR Factors in COPSAC2010 and COPSAC2000. Results of adjusted sub-analyses examining the associations between screen time and defined cardiometabolic risk factors, stratified by weekdays and weekends in the COPSAC2010 and COPSAC2000 cohorts. The associations are presented as estimates with 95% confidence intervals and corresponding p-values.

Outcome Variable	Phone Screen time [95% CI] p-value	TV Screen time [95% CI] p-value	Gaming Screen time [95% CI] p-value
Cardiometabolic Risk (Z-score)	0.15 [0.05 - 0.25] (p = 0.004)	0.14 [0.01 - 0.27] (p = 0.033)	0.15 [0.03 - 0.27] (p = 0.013)
Waist size (cm)	1.21 [0.35 - 2.06] (p = 0.006)	1.46 [0.34 - 2.58] (p = 0.011)	1.72 [0.71 - 2.73] (p = 0.001)
Systolic Blood Pressure (mmHg)	-0.18 [-0.96 - 0.6] (p = 0.646)	0.26 [-0.76 - 1.28] (p = 0.614)	1.95 [1.04 - 2.86] (p <0.001)
HDL Cholesterol (mmol/l)	-0.01 [-0.03 - 0.01] (p = 0.436)	-0.01 [-0.04 - 0.01] (p = 0.35)	-0.03 [-0.06 - -0.01] (p = 0.01)
Triglycerides (mmol/l)** Logged	0.05 [0.01 - 0.08] (p = 0.006)	0.03 [-0.01 - 0.08] (p = 0.18)	-0.01 [-0.05 - 0.03] (p = 0.699)
Glucose (mmol/l)	0.01 [-0.02 - 0.04] (p = 0.557)	-0.01 [-0.05 - 0.03] (p = 0.585)	0.04 [0 - 0.08] (p = 0.031)
HB1AC (mmol/mol)	-0.09 [-0.32 - 0.14] (p = 0.46)	-0.19 [-0.49 - 0.11] (p = 0.212)	0.04 [-0.23 - 0.3] (p = 0.776)
HOMA-IR	0.18 [0.02 - 0.34] (p = 0.029)	0.03 [-0.18 - 0.24] (p = 0.779)	0.1 [-0.09 - 0.28] (p = 0.308)
High sensitivity CRP (mg/L)	0.02 [-0.19 - 0.24] (p = 0.852)	-0.08 [-0.36 - 0.2] (p = 0.576)	-0.01 [-0.26 - 0.25] (p = 0.964)
GlycA (mmol/l)	0.01 [0 - 0.02] (p = 0.078)	0.01 [0 - 0.03] (p = 0.014)	0.01 [-0.01 - 0.02] (p = 0.373)
ApoB (g/l)	0.01 [0 - 0.03] (p = 0.037)	0.02 [0 - 0.04] (p = 0.031)	0 [-0.01 - 0.02] (p = 0.784)
NMR Cardiovascular Risk Score (Z-score)	0.08 [-0.01 - 0.17] (p = 0.092)	0.1 [-0.02 - 0.21] (p = 0.097)	0.05 [-0.06 - 0.16] (p = 0.399)

Table S7. Sub-analysis of Different Types of Screen Time Associations with CMR Factors in COPSAC2000 Cohort. Results of analyses examining the associations between different types of screen time (phone, TV, and gaming) and various cardiometabolic risk factors in the COPSAC2000 cohort. The associations are presented as estimates with 95% confidence intervals and corresponding p-values.

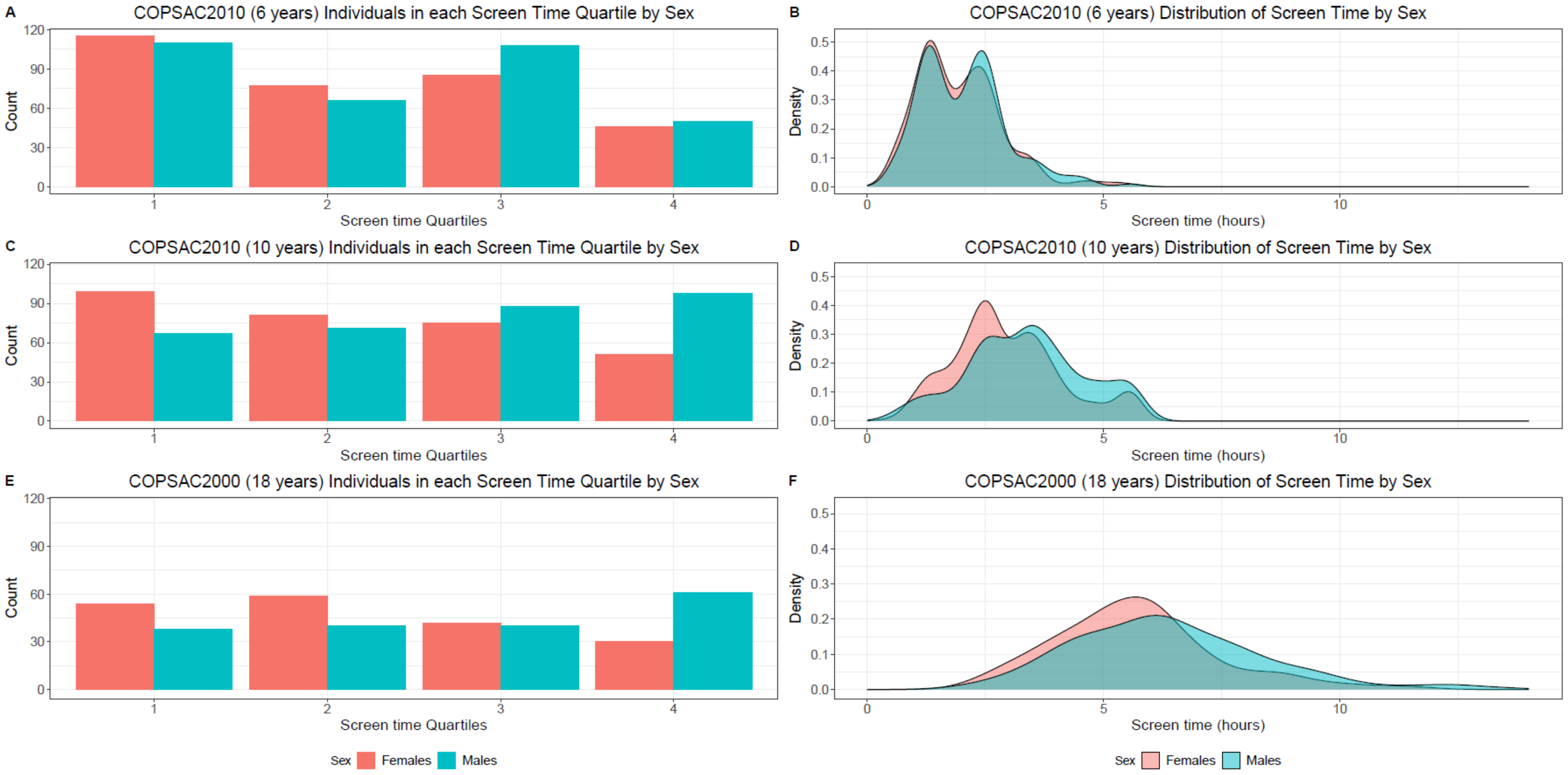

A
COPSAC2010 (6 years) Individuals in each Screen Time Quartile by Sex
Count
Screen time Quartiles
B
COPSAC2010 (6 years) Distribution of Screen Time by Sex
Density
Screen time (hours)
C
COPSAC2010 (10 years) Individuals in each Screen Time Quartile by Sex
Count
Screen time Quartiles
D
COPSAC2010 (10 years) Distribution of Screen Time by Sex
Density
Screen time (hours)
E
COPSAC2000 (18 years) Individuals in each Screen Time Quartile by Sex
Count
Screen time Quartiles
F
COPSAC2000 (18 years) Distribution of Screen Time by Sex
Density
Screen time (hours)
Sex Females Males
Sex Females Males

Figure S1. Distributions of Screen Time, Stratified by Sex, in COPSAC2010 and COPSAC2000. Distributions of screen time in the COPSAC2010 and COPSAC2000 cohorts. **Panel A and Panel C** display the quartile splits of screen time for the COPSAC2010 cohort at 6 years (n=657) and 10 years (n=630), respectively, further subdivided for males and females. **Panel E** illustrates the quartile splits of screen time for the COPSAC2000 cohort (n=364), with each quartile further split by sex. **Panels B, D, and F** show the density plots of the screen time distribution for the respective cohorts and time points, stratified by sex. The figure clearly illustrates marked sex differences in screen time within and between cohorts, as well as within each quartile of screen time.

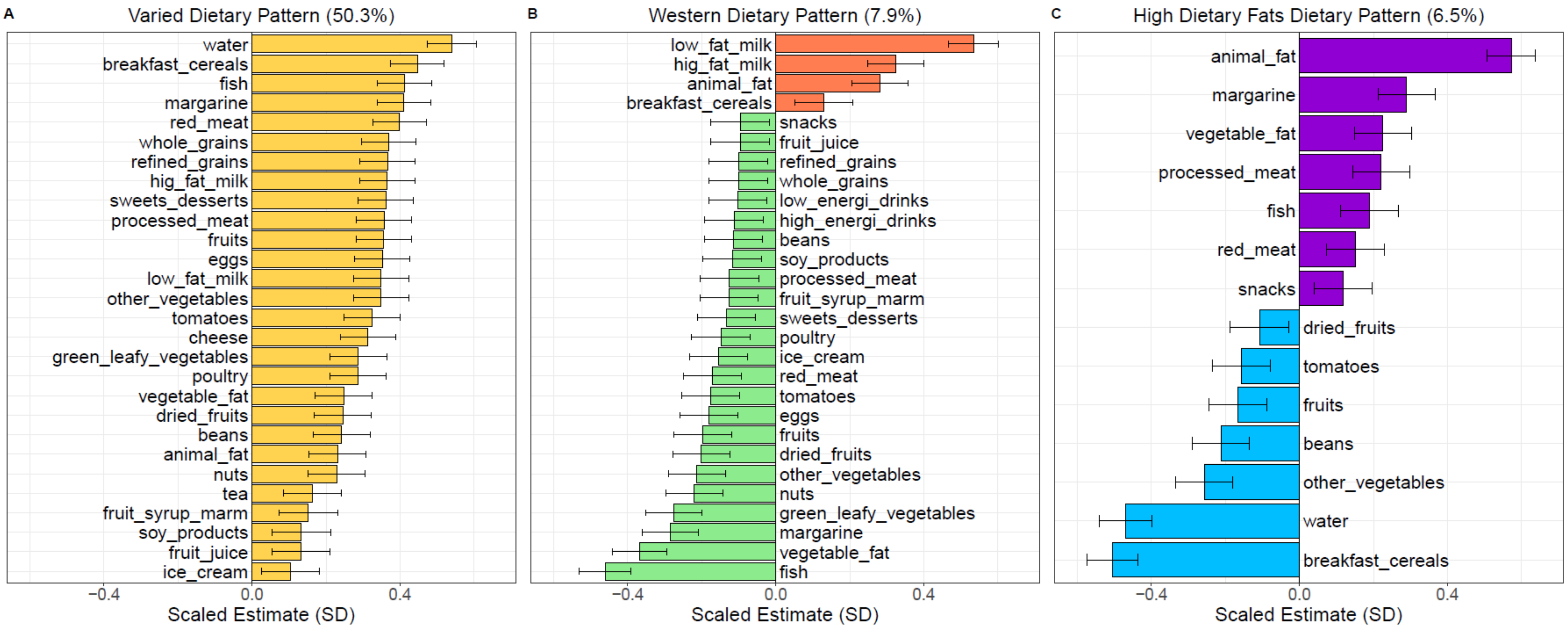

Figure S2. Associations of Principal Component 1 (`Varied Dietary Pattern`), Principal Component 2 (`Western Dietary Pattern`) and Principal Component 3 (`High Dietary Fats Dietary Pattern`) of Nutrient Constituents, assessed at 10 years with Food Frequency Questionnaires, with Food Frequency Questionnaires Derived Food Groups. This figure presents the associations between three dietary patterns derived from food frequency questionnaires at 10 years, and food frequency derived food groups. Panels A,B and C depict these associations for the Varied dietary pattern, Western dietary pattern and High dietary fats dietary pattern, respectively.

A B

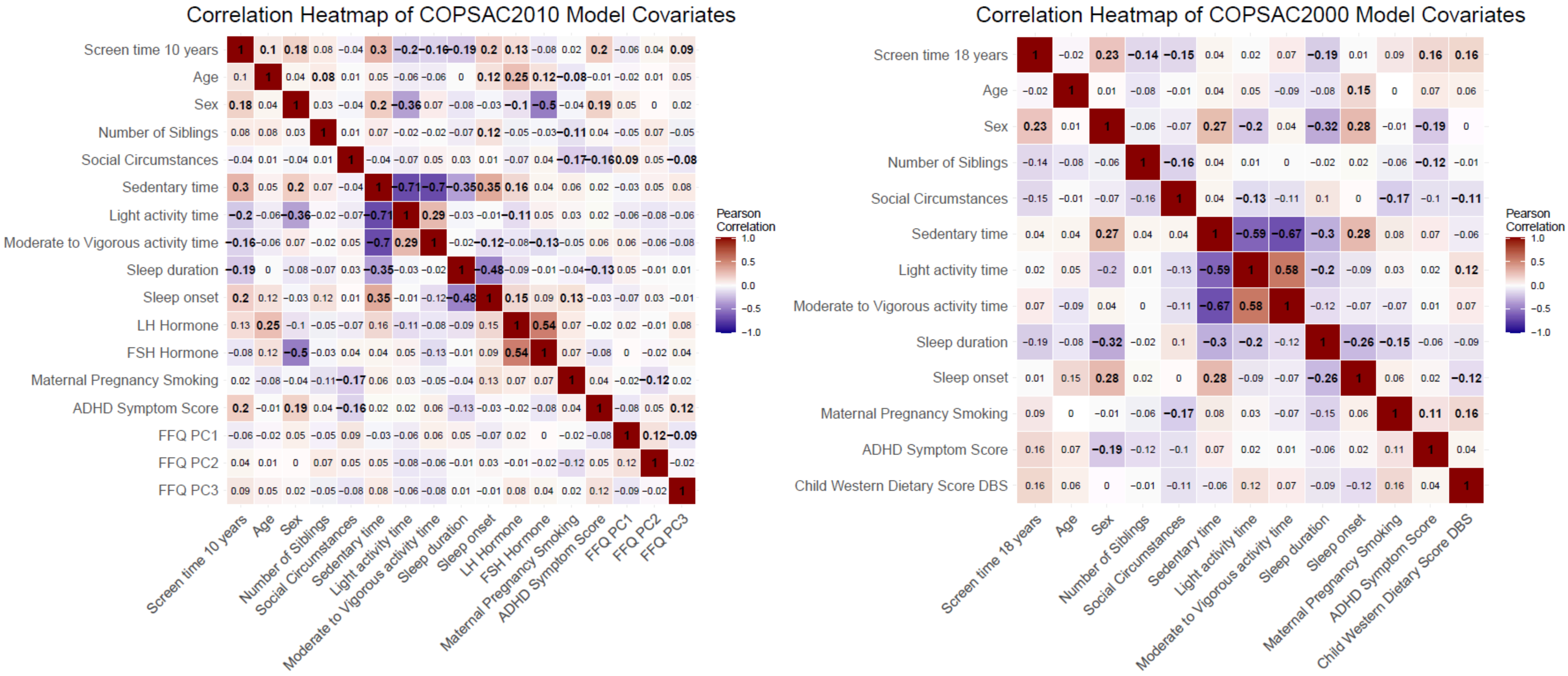
Correlation Heatmap of COPSAC2010 Model Covariates
Screen time 10 years
Age
Sex
Number of Siblings
Social Circumstances
Sedentary time
Light activity time
Moderate to Vigorous activity time
Sleep duration
Sleep onset
LH Hormone
FSH Hormone
Maternal Pregnancy Smoking
ADHD Symptom Score
FFQ PC1
FFQ PC2
FFQ PC3
Pearson Correlation
1.0
0.5
0.0
−0.5
−1.0
Correlation Heatmap of COPSAC2000 Model Covariates
Screen time 18 years
Age
Sex
Number of Siblings
Social Circumstances
Sedentary time
Light activity time
Moderate to Vigorous activity time
Sleep duration
Sleep onset
Maternal Pregnancy Smoking
ADHD Symptom Score
Child Western Dietary Score DBS
Pearson Correlation
1.0
0.5
0.0
−0.5
−1.0

Figure S3. Heatmap of Correlations between Screen Time and Model Covariates within Cohorts. Heatmap visualising the correlations between screen time and model covariates within each cohort. **Panel A** represents the COPSAC2010 cohort and **Panel B** represents the COPSAC2000 cohort. The colour intensity is proportional to the correlation coefficients, and significant coefficients ($p<0.05$) are in bold. This visualisation aids in understanding the relationships between screen time and various factors such as age, maternal smoking, physical activity, and sleep patterns, highlighting the multifactorial nature of screen time influences.

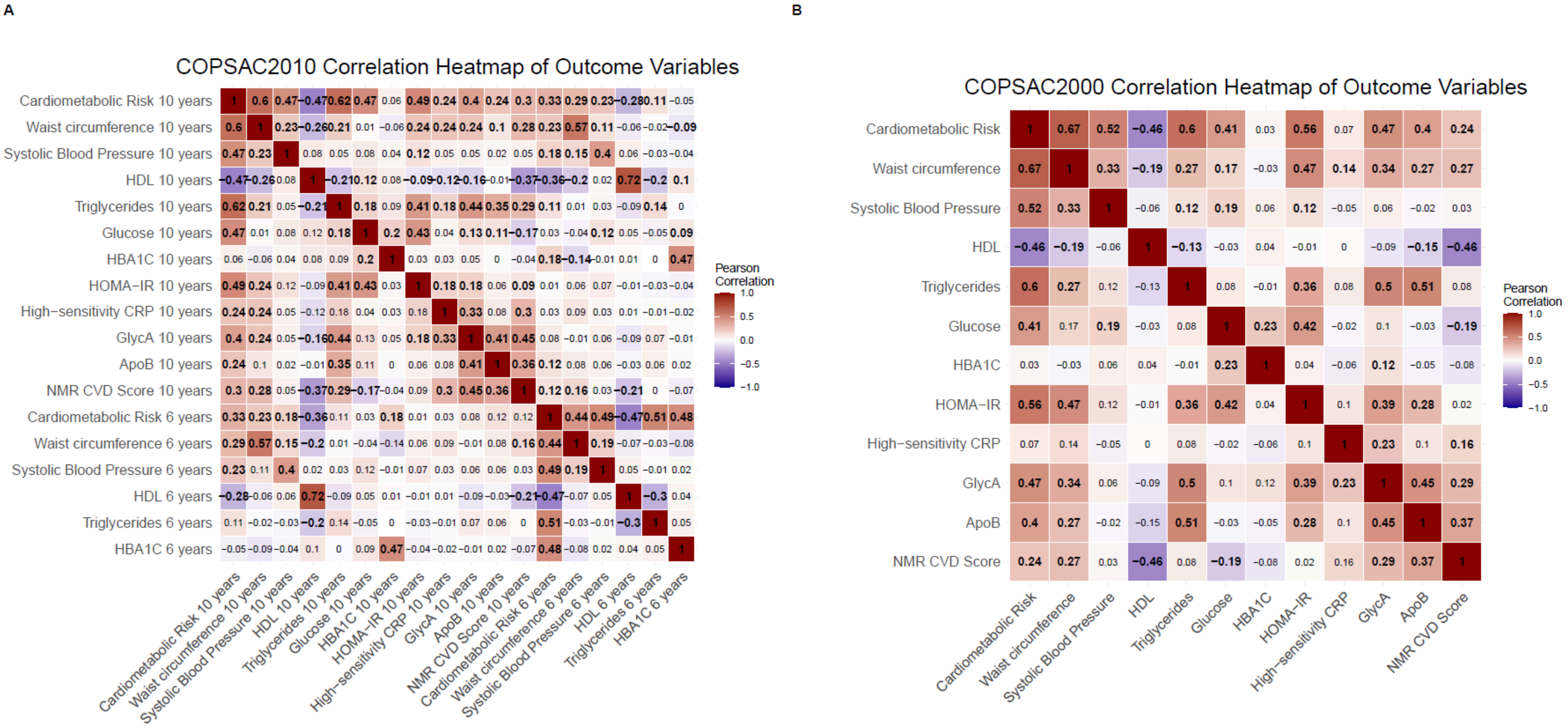
A
COPSAC2010 Correlation Heatmap of Outcome Variables
Cardiometabolic Risk 10 years
Waist circumference 10 years
Systolic Blood Pressure 10 years
HDL 10 years
Triglycerides 10 years
Glucose 10 years
HBA1C 10 years
HOMA−IR 10 years
High−sensitivity CRP 10 years
GlycA 10 years
ApoB 10 years
NMR CVD Score 10 years
Cardiometabolic Risk 6 years
Waist circumference 6 years
Systolic Blood Pressure 6 years
HDL 6 years
Triglycerides 6 years
HBA1C 6 years
Pearson Correlation
1.0
0.5
0.0
−0.5
−1.0
B
COPSAC2000 Correlation Heatmap of Outcome Variables
Cardiometabolic Risk
Waist circumference
Systolic Blood Pressure
HDL
Triglycerides
Glucose
HBA1C
HOMA−IR
High−sensitivity CRP
GlycA
ApoB
NMR CVD Score
Pearson Correlation
1.0
0.5
0.0
−0.5
−1.0

Figure S4. Heatmap of Correlations between Cardiometabolic Risk Factors within Cohorts. Heatmap visualising the correlations between various cardiometabolic risk factors within each cohort. **Panel A** represents the COPSAC2010 cohort with metabolic outcomes provided for both 6 years and 10 years. **Panel B** represents the COPSAC2000 cohort. The colour intensity is proportional to the correlation coefficients, and significant coefficients ($p<0.05$) are in bold. This visualisation provides a comprehensive view of the interrelationships between different cardiometabolic risk factors, including waist circumference, systolic blood pressure, triglycerides, glucose, and HDL, emphasising the complex interplay between these factors in determining overall cardiometabolic risk.

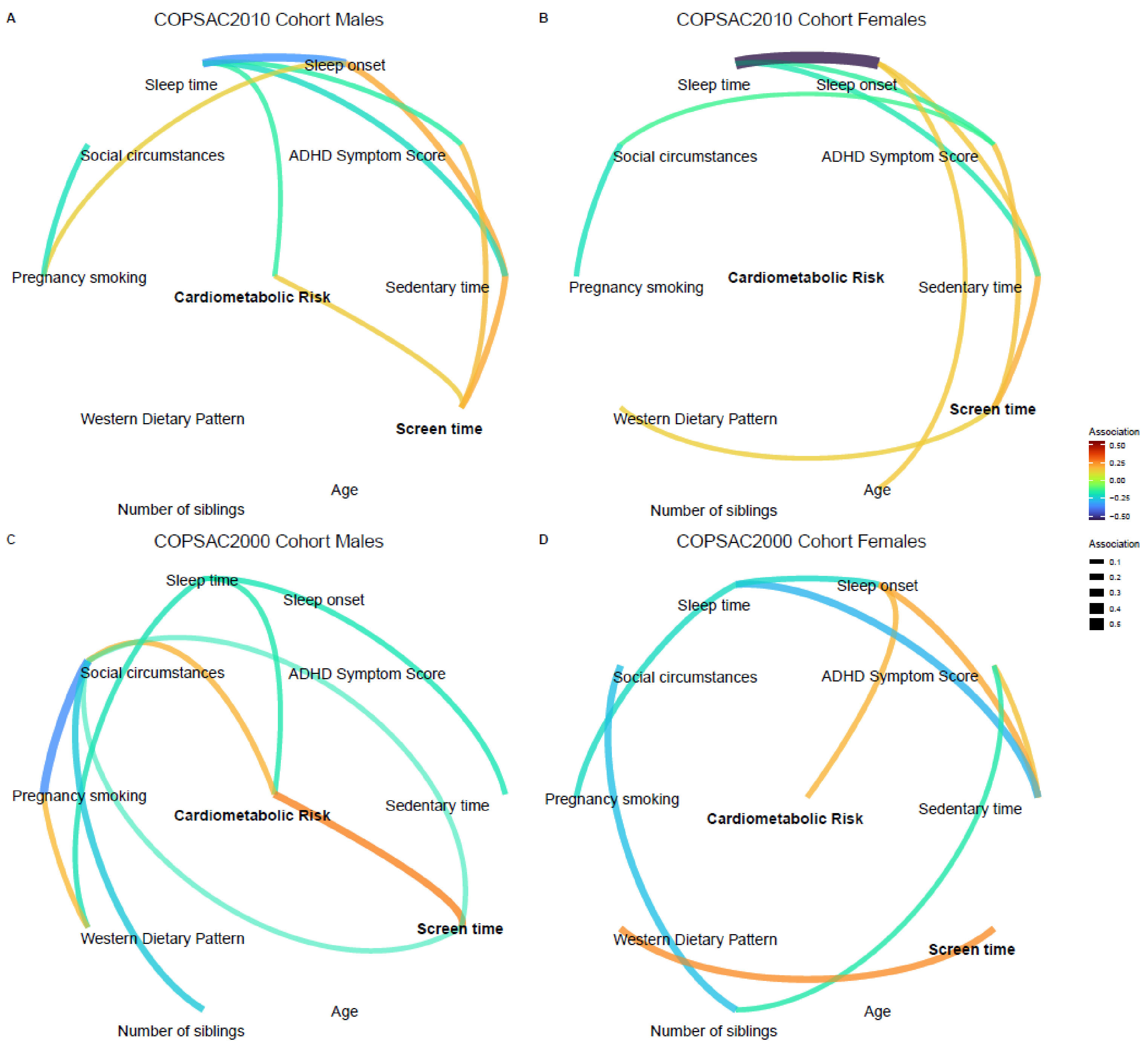

Figure S5. Gaussian Graphical Models Depicting the Integrated Relationships between Cardiometabolic Risk, Screen Time, and Other Covariates, Stratified by Sex, in COPSAC2010 and COPSAC2000. Graphical models illustrate relationships (95% CI) between screen time,cardiometabolic risk, and model covariates. **Panels A and B** represent COPSAC2010 stratified by sex, whereas **Panels C and D** represent COPSAC2000 stratified by sex.

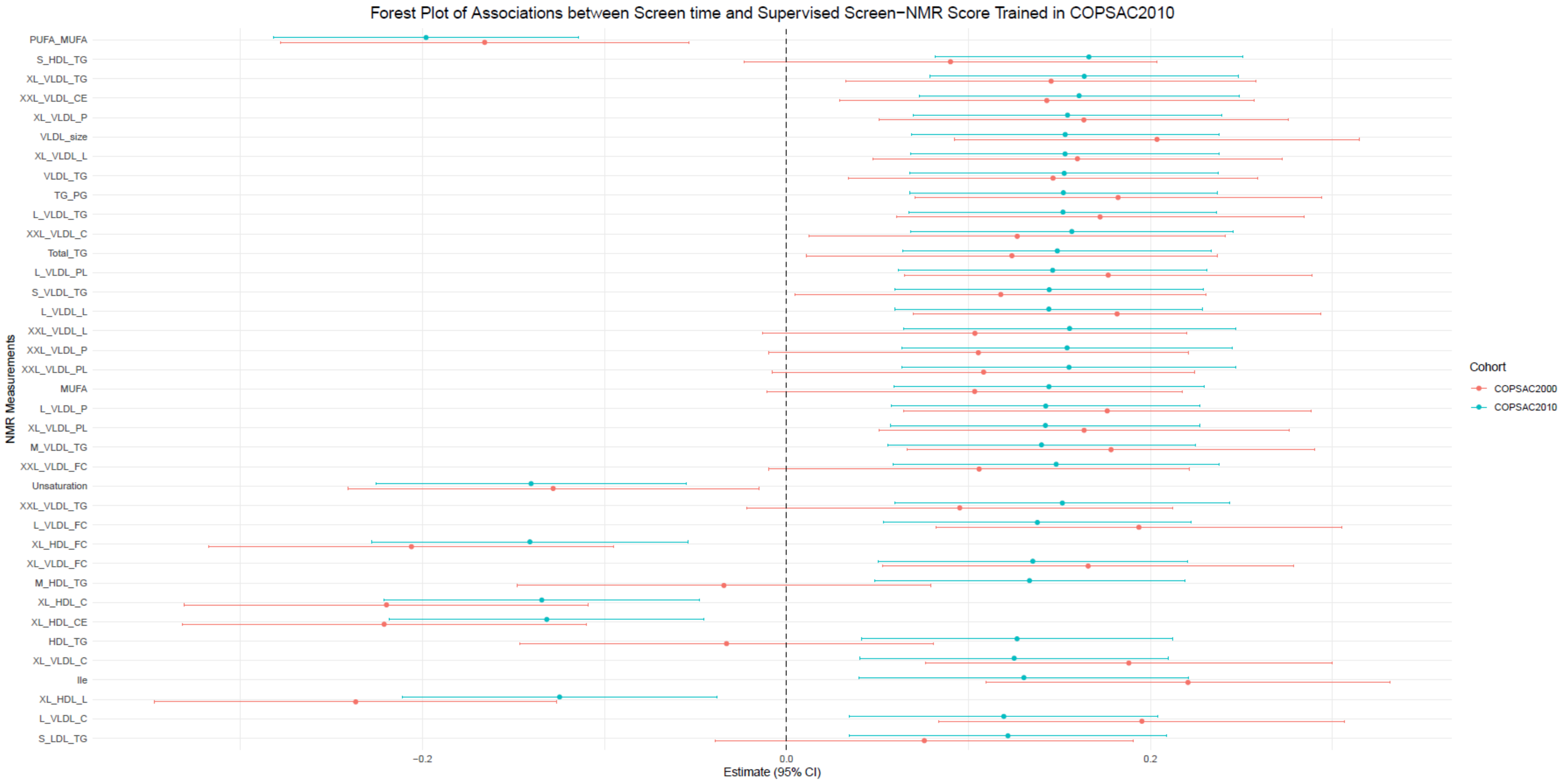

Forest Plot of Associations between Screen time and Supervised Screen−NMR Score Trained in COPSAC2010
PUFA_MUFA
S_HDL_TG
XL_VLDL_TG
XXL_VLDL_CE
XL_VLDL_P
VLDL_size
XL_VLDL_L
VLDL_TG
TG_PG
L_VLDL_TG
XXL_VLDL_C
Total_TG
L_VLDL_PL
S_VLDL_TG
L_VLDL_L
XXL_VLDL_L
XXL_VLDL_P
XXL_VLDL_PL
MUFA
L_VLDL_P
XL_VLDL_PL
M_VLDL_TG
XXL_VLDL_FC
Unsaturation
XXL_VLDL_TG
L_VLDL_FC
XL_HDL_FC
XL_VLDL_FC
M_HDL_TG
XL_HDL_C
XL_HDL_CE
HDL_TG
XL_VLDL_C
Ile
XL_HDL_L
L_VLDL_C
S_LDL_TG
NMR Measurements
−0.2
0.0
0.2
Estimate (95% CI)
Cohort
COPSAC2000
COPSAC2010

Figure S6. Forest Plot of Associations between Screen Time and Selected NMR Metabolites Identified in COPSAC2010 Forest plot illustrating the associations between z-scored screen time within each cohort and selected NMR metabolites from the supervised sparse partial least squares model trained in COPSAC2010. The model considered 173 metabolic biomarkers and retained 37 screen time-associated biomarkers. This figure depicts the strength and direction of these associations, highlighting the robust predictive capability of the identified metabolic disturbances related to screen time across both COPSAC2010 and COPSAC2000 cohorts.

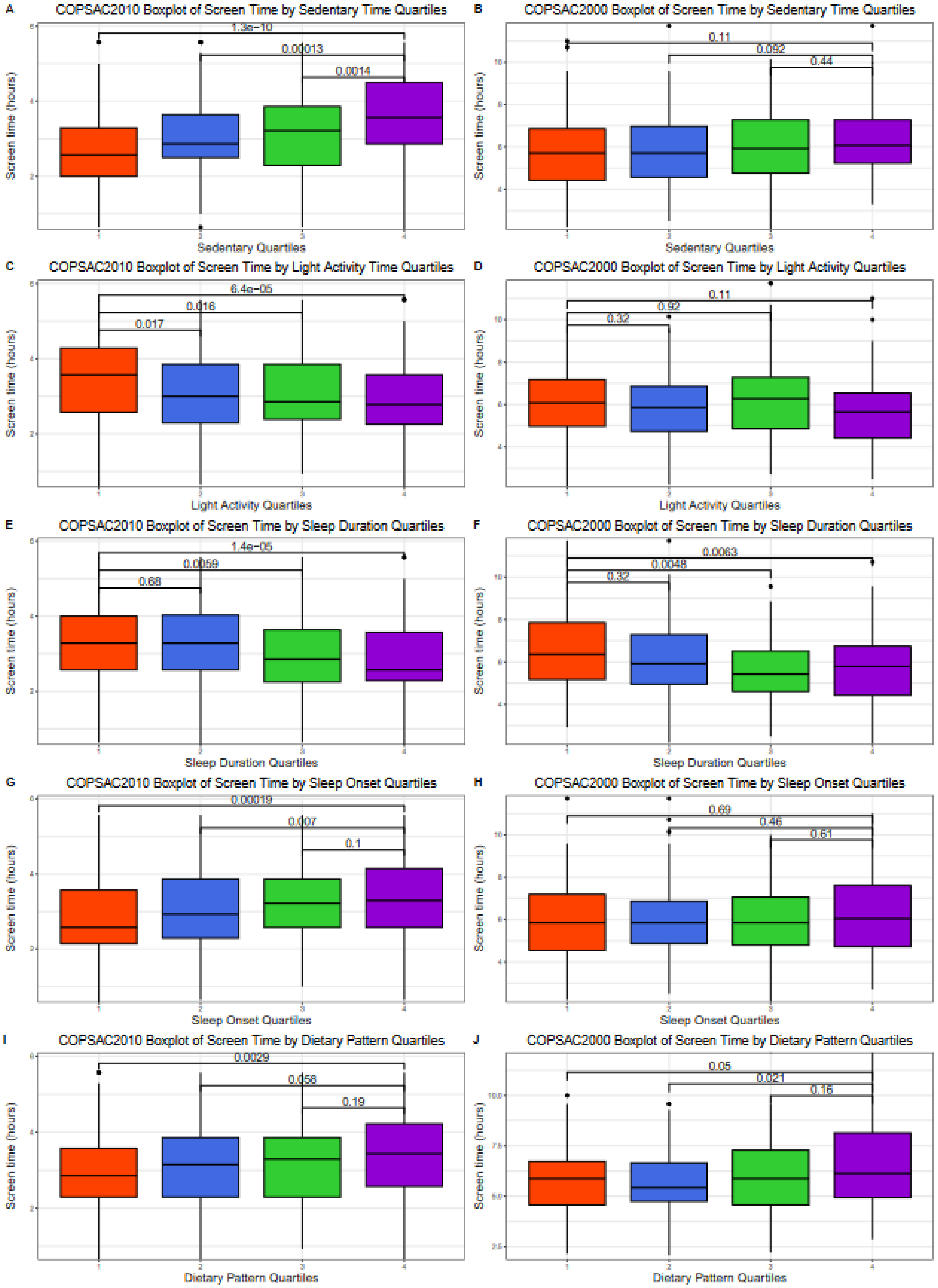
A
COPSAC2010 Boxplot of Screen Time by Sedentary Time Quartiles
1.3e−10
0.00013
0.0014
Screen time (hours)
Sedentary Quartiles
B
COPSAC2000 Boxplot of Screen Time by Sedentary Time Quartiles
0.11
0.092
0.44
Screen time (hours)
Sedentary Quartiles
C
COPSAC2010 Boxplot of Screen Time by Light Activity Time Quartiles
6.4e−05
0.016
0.017
Screen time (hours)
Light Activity Quartiles
D
COPSAC2000 Boxplot of Screen Time by Light Activity Quartiles
0.11
0.92
0.32
Screen time (hours)
Light Activity Quartiles
E
COPSAC2010 Boxplot of Screen Time by Sleep Duration Quartiles
1.4e−05
0.0059
0.68
Screen time (hours)
Sleep Duration Quartiles
F
COPSAC2000 Boxplot of Screen Time by Sleep Duration Quartiles
0.0063
0.0048
0.32
Screen time (hours)
Sleep Duration Quartiles
G
COPSAC2010 Boxplot of Screen Time by Sleep Onset Quartiles
0.00019
0.007
0.1
Screen time (hours)
Sleep Onset Quartiles
H
COPSAC2000 Boxplot of Screen Time by Sleep Onset Quartiles
0.69
0.46
0.61
Screen time (hours)
Sleep Onset Quartiles
I
COPSAC2010 Boxplot of Screen Time by Dietary Pattern Quartiles
0.0029
0.058
0.19
Screen time (hours)
Dietary Pattern Quartiles
J
COPSAC2000 Boxplot of Screen Time by Dietary Pattern Quartiles
0.05
0.021
0.16
Screen time (hours)
Dietary Pattern Quartiles

Figure S7. Associations of Lifestyle Factors with Screen Time in COPSAC2010 and COPSAC2000. Associations between screen time and lifestyle factors, including sedentary and light activity time, sleep duration and onset, and dietary patterns. **Panels A and B** depict the relationship between screen time and sedentary time, **Panels C and D** show the relationship with light activity time, **Panels E and F** illustrate the relationship with sleep duration, **Panels G and H** present the relationship with sleep onset, and **Panels I and J** depict the relationship with a Western dietary pattern. The left panels (**A, C, E, G, I**) represent the COPSAC2010 cohort, while the right panels (**B, D, F, H, J**) represent the COPSAC2000 cohort. Annotated values are Wilcoxon p-values comparing the most sedentary, least light activity, least sleep duration, latest sleep onset, and most Western dietary pattern metabolome scores with the other quartiles.

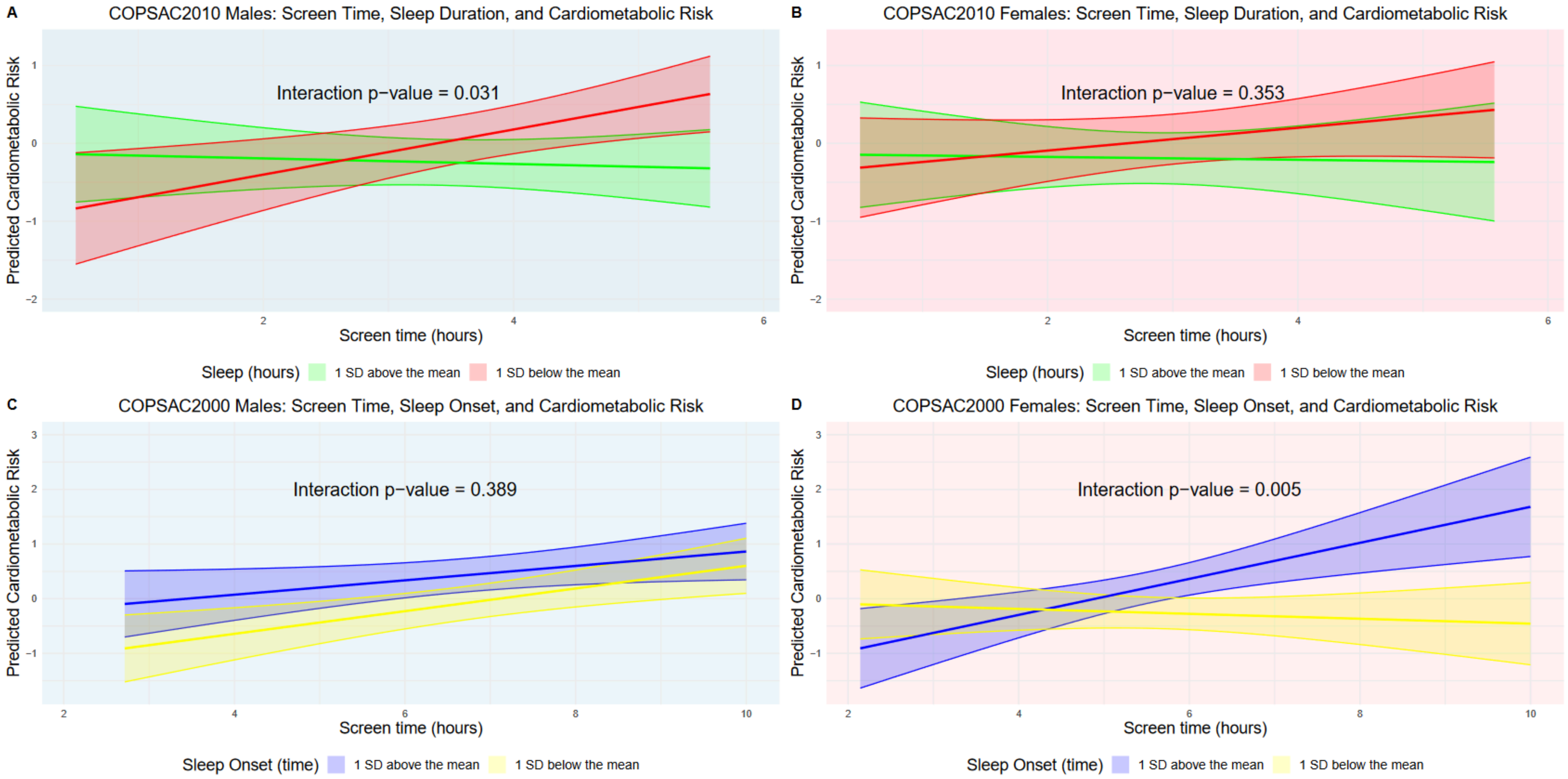
A
COPSAC2010 Males: Screen Time, Sleep Duration, and Cardiometabolic Risk
Interaction p−value = 0.031
Predicted Cardiometabolic Risk
Screen time (hours)
Sleep (hours)
1 SD above the mean
1 SD below the mean
B
COPSAC2010 Females: Screen Time, Sleep Duration, and Cardiometabolic Risk
Interaction p−value = 0.353
Predicted Cardiometabolic Risk
Screen time (hours)
Sleep (hours)
1 SD above the mean
1 SD below the mean
C
COPSAC2000 Males: Screen Time, Sleep Onset, and Cardiometabolic Risk
Interaction p−value = 0.389
Predicted Cardiometabolic Risk
Screen time (hours)
Sleep Onset (time)
1 SD above the mean
1 SD below the mean
D
COPSAC2000 Females: Screen Time, Sleep Onset, and Cardiometabolic Risk
Interaction p−value = 0.005
Predicted Cardiometabolic Risk
Screen time (hours)
Sleep Onset (time)
1 SD above the mean
1 SD below the mean

Figure S8. Sex-Stratified Analyses of Interactions between Screen Time and Sleep. Results of sex-stratified analyses examining potential sex differences in the interactions between screen time and sleep duration/onset. **Panel A** represents the interaction between screen time and sleep duration in COPSAC2010 males, showing a pronounced negative trend (p=0.006) **Panel D** represents the interaction between screen time and sleep onset in COPSAC2000 females, showing a significant positive trend (p=0.005). These findings highlight the potential sex differences in the modulating effects of sleep factors on the relationship between screen time and cardiometabolic risk.
